# Compensatory mechanisms in resistant *Anopheles gambiae* AcerKis and KdrKis neurons modulate insecticide-based mosquito control

**DOI:** 10.1038/s42003-021-02192-0

**Published:** 2021-06-02

**Authors:** Stéphane Perrier, Eléonore Moreau, Caroline Deshayes, Marine El-Adouzi, Delphine Goven, Fabrice Chandre, Bruno Lapied

**Affiliations:** 1 Univ Angers, INRAE, SIFCIR, SFR QUASAV, Angers, France; 2 MIVEGEC, UMR IRD 224-CNRS 5290-Université de Montpellier, 911 avenue Agropolis, Montpellier, Cedex 05 France

**Keywords:** Neuroscience, Ion channels in the nervous system

## Abstract

In the malaria vector *Anopheles gambiae*, two point mutations in the acetylcholinesterase (*ace-1*^*R*^) and the sodium channel (*kdr*^*R*^) genes confer resistance to organophosphate/carbamate and pyrethroid insecticides, respectively. The mechanisms of compensation that recover the functional alterations associated with these mutations and their role in the modulation of insecticide efficacy are unknown. Using multidisciplinary approaches adapted to neurons isolated from resistant *Anopheles gambiae* AcerKis and KdrKis strains together with larval bioassays, we demonstrate that nAChRs, and the intracellular calcium concentration represent the key components of an adaptation strategy ensuring neuronal functions maintenance. In AcerKis neurons, the increased effect of acetylcholine related to the reduced acetylcholinesterase activity is compensated by expressing higher density of nAChRs permeable to calcium. In KdrKis neurons, changes in the biophysical properties of the L1014F mutant sodium channel, leading to enhance overlap between activation and inactivation relationships, diminish the resting membrane potential and reduce the fraction of calcium channels available involved in acetylcholine release. Together with the lower intracellular basal calcium concentration observed, these factors increase nAChRs sensitivity to maintain the effect of low concentration of acetylcholine. These results explain the opposite effects of the insecticide clothianidin observed in AcerKis and KdrKis neurons in vitro and in vivo.

## Abstract (web summary)

Perrier et al. examine the neurons of mosquitoes following the development of insecticide resistance-associated point mutations in the voltage-gated sodium channels and AChE1 genes for two resistant strains, KdrKis and AcerKis. Their results show that nAChRs and the intracellular calcium concentration provide a compensatory mechanism for AcerKis and KdrKis neurons to insecticide exposure.

## Introduction

Vector-borne diseases account for an important number of human illnesses. They represent 17% of the global infectious diseases, causing almost one million deaths each year^1^. Based on the sixth Global Fund replenishment conference held in 2019, malaria is one of the three most dangerous infectious disease worldwide. It accounts for a total of about 405,000 deaths and 228 million contamination cases each year, putting half of the world’s population at risk in either Africa, South-East Asia, Eastern Mediterranean, Western Pacific or Americas^2^. Mosquitoes belonging to *Anopheles gambiae s.l*. complex of species are major vectors of malaria in sub Saharan Africa. Prevention of malaria transmission is largely implemented by the use of insecticide-treated bed nets (ITNs) and/or the development of strategies in insecticide-based mosquito control to prevent mosquito biting at the individual and household levels. In this context, insecticides are one of the important arsenals in the fight against mosquito vectors of malaria to save hundreds of millions of lives^3^. However, the development of insecticide resistance in vector mosquitoes, may cause control failure, allowing a resurgence of malaria in endemic countries^4^.

Effective insecticide resistance management (IRM) is difficult and detecting resistance before it becomes an operational problem is a complex challenge. Insecticide resistance is a rapid adaptive response of mosquito populations due to many selection pressures exerted by human activities in mosquitoes. Multiple resistance mechanisms occurring in mosquitoes are an inherited characteristic involving several physiological and/or behavioral changes. Among classical examples of resistance mechanisms, an overexpression of several enzyme families such as cytochromes P450, esterases and glutathione-S-transferases is well described^4–7^. In addition, mosquitoes acquire target site insensitivity through mutations (e.g., *kdr*, *super-kdr*, *rdl*, *ace-1*^*R*^) within structural genes that reduce the binding of the insecticide^4,5,7–10^. The reduced penetration of insecticides is also another process now considered as a resistance mechanism in mosquitoes^4,11,12^. Furthermore, insecticide resistance mechanisms can also be referred as behavioral changes in response to insecticide exposure allowing mosquitoes to overcome management tactics^4,13–17^. Today, new additional insecticide resistance mechanisms involving the implication of unsuspected proteins identified in *Anopheles gambiae* also contribute to the resistance phenotype^7,18–21^. Using a meta-analysis approach of transcriptomic data from *Anopheles gambiae* populations resistant to the commonly used pyrethroid insecticides, novel multiple up-regulated gene families coding for insecticide-binding proteins have been characterized. This includes members of hexamerins and/or α-crystallins^22^, up-regulated in response to deltamethrin exposure that reduce mosquito mortality following deltamethrin treatment^20^. A whole-genome microarray approach indicates that overexpression of genes encoding salivary gland proteins can be closely associated with insecticide resistance in *Anopheles gambiae*^19^. Another mechanism recently identified, involves mosquito sensory appendage protein, (e.g., SAP2), a member of chemosensory protein family known to be implicated in the transport of hydrophobic compounds^23^. They participate in the development of pyrethroid resistance through the binding of insecticides at the first point of mosquito contact with bed nets^21^.

The outcomes of these studies highlight the complexity of mechanisms involved in insecticide resistance. They also point out a research area that has never been investigated. It concerns the characterization of unsuspected physiological cellular and/or molecular events, which emerge as the consequences of the resistance mechanisms. These events, acting as compensatory mechanisms to strengthen physiological functions, may undoubtedly impact the effectiveness of the IRM strategies.

To the best of our knowledge, in this study we investigate in mosquitoes the neuronal compensatory mechanisms following the development of resistance-associated point mutations in the voltage-gated sodium channels^5,24^ and AChE1^25,26^ genes characterized in two strains of *Anopheles gambiae* resistant to two distinct classes of insecticides, the pyrethroid/DDT-resistant strain, named KdrKis (*kdr*^*R*^, L1014F) and the organophosphate/carbamate-resistant strain, named AcerKis (*ace-1*^*R*^, G119S). We use multidisciplinary approaches adapted to isolated *Anopheles gambiae* neurons^27^ to characterize changes in the expression and pharmacological profile of neuronal nicotinic acetylcholine receptors (nAChRs) following the mutations G119S in AcerKis strain and L1014F in KdrKis strain compared to the susceptible laboratory reference *Anopheles gambiae* Kis strain. Parallel bioassays have also been conducted on AcerKis and KdrKis larvae to establish correlation between in vivo and in vitro studies. Firstly, we report that higher nAChRs density associated with reduced activity of AChE1 (a key enzyme in the nervous system, which terminates nerve impulses by catalysing the hydrolysis of neurotransmitter acetylcholine) is linked to the mutation G119S in neurons from Acerkis strain. Secondly, the modification of biophysical properties of the voltage-gated sodium channel (known to play an essential role in the initiation and propagation of action potentials in neurons) and studied for the first time in neurons from KdrKis strain, impacts nAChRs sensitivity to acetylcholine. Thirdly, we indicate that these unexpected compensatory physiological mechanisms, which both affect the cholinergic system, can modify nAChRs sensitivity to the recommended insecticide clothianidin, in vitro and in vivo. Our results demonstrate that highly complex compensatory mechanisms related to point mutations are essential to be understood for the development of insecticide-based strategies used for mosquito control.

## Results

### Effect of acetylcholine on isolated neurons from *Anopheles gambiae* Kis, AcerKis and KdrKis strains

**Fig. 1:**
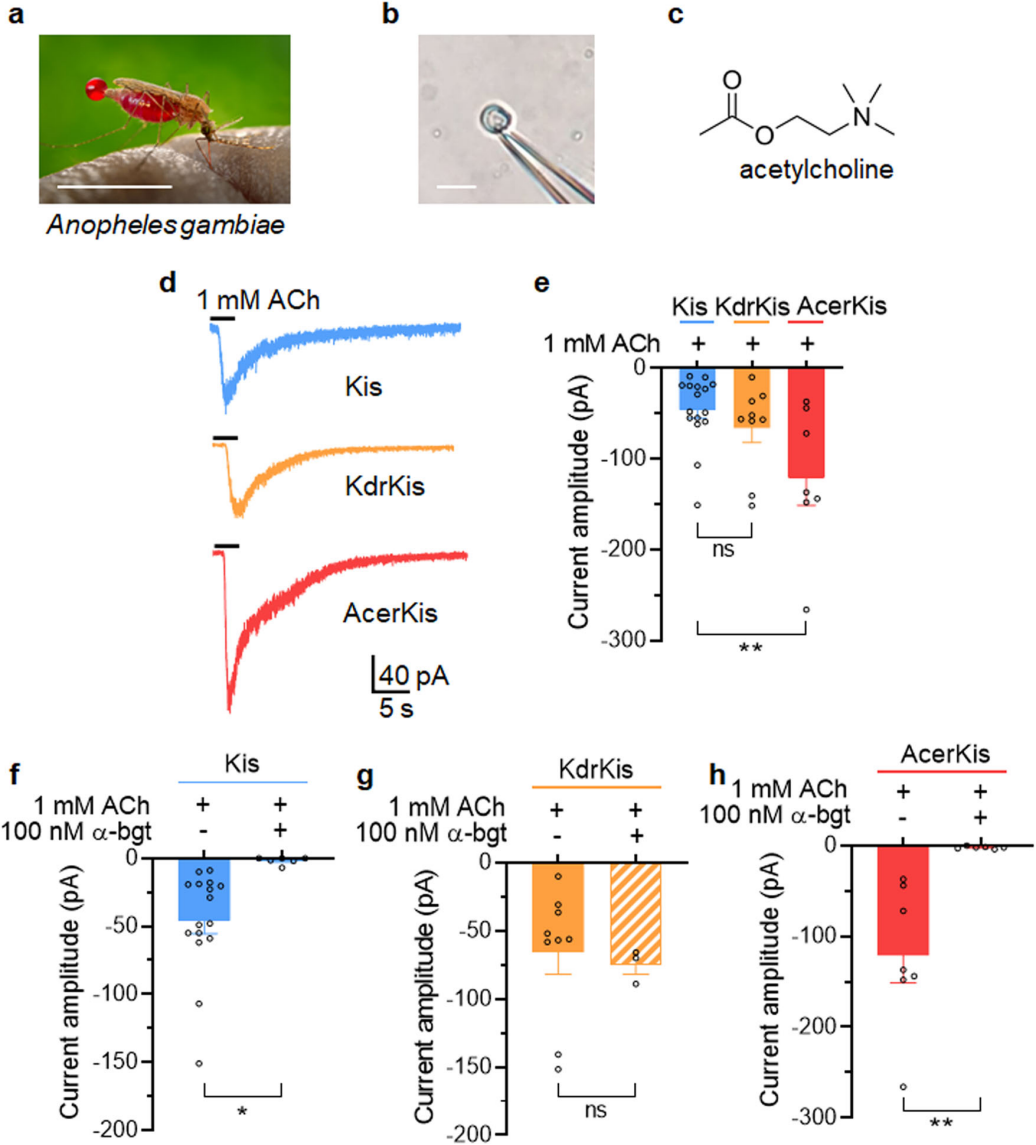
Acetylcholine induces transient inward currents in isolated neurons from *Anopheles gambiae*, Kis, AcerKis and KdrKis strains. **a** Photograph of the lateral view of a female *Anopheles gambiae*. **b** Light micrograph of the whole cell patch-clamp technique adapted on the isolated adult mosquito neuron cell body. **c** The chemical structure of the natural neurotransmitter acetylcholine. **d** Typical examples of steady-state recordings of acetylcholine (ACh)-induced inward currents obtained in whole-cell voltage-clamp mode at a steady-state holding potential of −50 mV. Pulse of ACh (1 mM, 3 s in duration) was applied onto the isolated neuron cell body from three *Anopheles gambiae* strains, Kis, KdrKis and AcerKis, as indicated below each current trace. **e** Histogram summarizing the ACh-induced current amplitudes recorded at a holding potential of −50 mV in three strains of *Anopheles gambiae* isolated neurons indicated above each bar. Bars represent mean ± S.E.M. (*n* = 7–16); Statistical test used was Student unpaired *t*-test, **p < 0.01; ns, non-significant. **f**–**h** Comparative histograms illustrating the effect of the nicotinic receptor antagonist α-bungarotoxin (α-bgt; 100 nM) on the ACh-induced inward current amplitudes, recorded at a holding potential of −50 mV in isolated neurons from mosquito strains Kis (**f**), KdrKis (**g**) and Acerkis (**h**). Bars represent mean ± S.E.M. (*n* = 4–6); Statistical test used was Student unpaired *t*-test, **p < 0.01; *p < 0.05; ns, non-significant. Scale bar 1 cm (**a**) and 10 µm (**b**). The number of experiments (*n*) are biologically independent samples. The image presented in **a** is in the public domain and thus free of any copyright restrictions (CDC/James Gathany, 2014).

Whole-cell patch clamp technique, adapted on isolated neurons^27^ from *Anopheles gambiae* (Fig. 1a, b), was used to study the electrophysiological properties of the ACh-induced inward currents (Fig. 1c) mediated by nAChRs, in the presence of 100 nM atropine to block muscarinic acetylcholine receptors (mAChRs). Under steady-state voltage-clamp condition (holding potential of −50mV), pulse application of ACh (1 mM, 3 s in duration) onto the soma of isolated neuron cell bodies form Kis, KdrKis and AcerKis strains, induced transient inward currents which vary in amplitude (Fig. 1d). As illustrated in Fig. 1e, the mean current amplitudes were −45.8 ± 9.5pA (*n* = 16), −65.8 ± 16.8pA (*n* = 10) and −121.1 ± 30pA (*n* = 7) for Kis, KdrKis and AcerKis neurons, respectively. Although current amplitudes appeared to be statistically non-significant between Kis and KdrKis neurons, peak current amplitudes were on average ~2.5 larger in AcerKis than in Kis neurons. Bath application of the nAChR antagonist α-bungarotoxin (α-bgt, 100 nM) completely inhibited the inward current induced by 1 mM ACh in both Kis and AcerKis neurons (*n* = 4–6). Interestingly, α-bgt did not produce any effect on ACh current amplitude in KdrKis neurons (Fig. 1f, g, h). Such inability of α-bgt to bind to neuronal nAChRs, involving specific residues that render nAChR insensitive to α-bgt binding, have already been described in vertebrates but also in insects^28,29^. These results suggest the existence of different types of nAChRs and indicate that the resistance-associated point mutations observed in AcerKis and KdrKis strains differentially affect the cholinergic system involving nAChRs.

### Increased density of nAChRs in neurons isolated from AcerKis strain

**Fig. 2:**
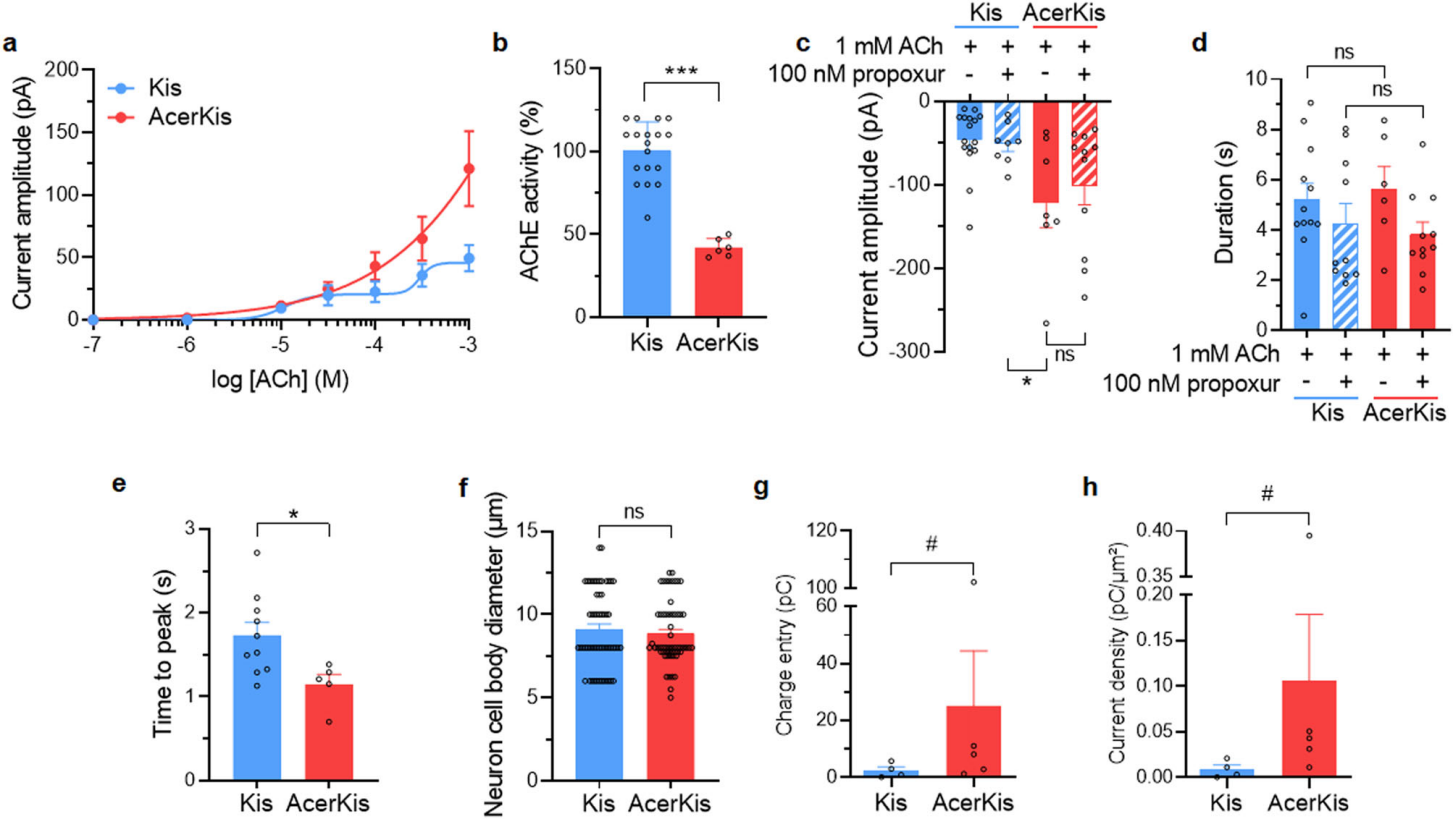
Acetylcholine induces higher current amplitude in neurons isolated from *Anopheles gambiae* strain AcerKis. **a** Superimposed semilogarithmic dose-response curves for the ACh-induced currents recorded at a holding potential of −50 mV in isolated neurons from mosquito strains Kis (*n* = 5–15) and AcerKis (*n* = 3–14), as indicated in the graph. Data are mean ± S.E.M. **b** Comparative histogram illustrating the % acetylcholinesterase (AChE) activity determined spectrophotometrically in neurons isolated from *Anopheles gambiae* Kis (*n* = 18) and AcerKis (*n* = 6) strains. Bars represent mean ± S.D; Statistical test used was Student unpaired *t*-test, ***p < 0.001. Note the strong reduction in AChE activity measured in AcerKis neurons. **c**, **d** Histograms summarizing the effects of the carbamate insecticide, propoxur (100 µM), known to inhibit acetylcholinesterase, on both current amplitudes (**c**) and durations (**d**) evoked by ACh (1 mM, 3 s in duration). The current duration was measured at 50% of the amplitude. Bars represent mean ± S.E.M. (*n* = 6–16); Statistical test used was Student unpaired *t*-test, *p < 0.05; ns, non-significant. **e** The time to peak of the ACh-induced inward current recorded in AcerKis neurons was compared to that of measured in Kis neurons. Bars represent mean ± S.E.M. (*n* = 5–10); Statistical test used was Student unpaired *t*-test, *p < 0.05. **f** Comparison of cell body diameters between neurons isolated from Kis (*n* = 56) and AcerKis (*n* = 57) strains. Bars represent mean ± S.E.M. Statistical test used was the Mann–Whitney test, ns, non-significant. **g**, **h** Comparative histograms illustrating the charge density (**g**), calculated from the integral of the current according to the Eq. (3) and the current density (**h**) measured from the ACh-evoked inward current recorded in Kis and AcerKis neurons. Bars represent mean ± S.E.M (*n* = 4–5), Statistical test used was the Mann–Whitney test ^#^*p* < 0.02. In all cases, the number of experiments (*n*) are biologically independent samples.

**Fig. 3:**
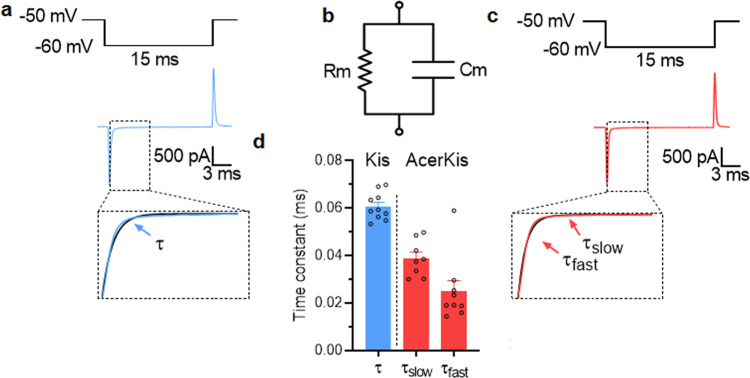
Total membrane capacitance measurement in isolated neurons from Kis and AcerKis *Anopheles gambiae* strains. **a**–**c** Typical examples of the capacitive current transients were recorded under voltage-clamp condition at a holding potential of −50 mV following a hyperpolarized voltage pulse according to the protocols indicated above each trace in Kis (**a**) and AcerKis (**c**) neurons. (**b**) represents the reduced equivalent circuit of the neuron cell body plasma membrane obtained by combining a fixed capacitance (Cm) in parallel with ion-specific pathway (Rm). The decay phase of the transient was well-fitted with a single exponential in Kis neurons whereas two exponential functions were used to fit the decay phase of the transient in AcerKis neurons giving fast and slow time constants (see text for details). **d** The corresponding comparative histogram illustrates the mean values of the time constants calculated for Kis and AcerKis neurons. Bars represent mean ± S.E.M. (*n* = 9–10). The number of experiments (*n*) are biologically independent samples.

To further study the origin of the high current amplitude observed in neurons isolated from AcerKis strains, the mean values of the peak inward current amplitude were plotted against the logarithm of the non-cumulative concentration of ACh. Pulse applications of ACh (3 s in duration), performed at concentrations ranging from 100 nM to 1 mM, induced a biphasic dose-response curve in Kis neurons (Fig. 2a; *n* = 5–15). The data were best-fitted through the mean data points according to the Eq. (2). An apparent maximum was obtained between 30 µM and 100 µM of ACh, but it was only a plateau between the two parts of the curve. For higher concentrations than 100 µM, ACh-induced current further increased before reaching maximum amplitude at approximately 1 mM. For comparison, the effect of ACh plotted as a semi-logarithmic function was best represented by a monophasic curve fitted by the Hill Eq. (1) in AcerKis neurons. Current amplitudes were enhanced from 100 µM ACh compared to those recorded in Kis neurons (Fig. 2a; *n* = 3–14). Previous findings have reported that AChE enzymatic activity was reduced in mosquito AcerKis strains^30,31^. Because AChE is one of the most crucial enzymes that hydrolyzes the neurotransmitter ACh, additional experiments were designed to determine if the reduction of AChE enzymatic activity was correlated with the high current amplitude recorded in isolated AcerKis neurons. Using biochemical assay of AChE enzyme activity performed on isolated neurons, the present study shows that AcerKis neurons also displayed a significant reduction in AChE enzymatic activity (from 100 ± 17%, *n* = 18, to 42 ± 5%, *n* = 6, in Kis and AcerKis neurons, respectively; Fig. 2b). In accordance with a typical reduction of AChE enzymatic activity, both amplitude and duration of the inward current elicited by ACh should increase, as predicted. However, experiments performed with propoxur (100 nM), the anticholinesterasic carbamate insecticide, did not produce any significant effect on both amplitude and duration of the ACh-induced current recorded in Kis neurons compared to AcerKis neurons (Fig. 2c, d). In other words, the lack of effect of propoxur on the current amplitude in Kis neurons is consistent with the fact that the reduction of AChE enzymatic activity, by itself, was not responsible for the high ACh-induced current amplitudes recorded in AcerKis neurons. From these results, it is tempting to consider an alternative hypothesis that may account for the high current amplitude observed in AcerKis neurons. Modifying the density of nAChRs in AcerKis neurons will undoubtedly change their activity pattern. To investigate this hypothesis, the time to peak of ACh-induced inward current was first measured to see whether it was modified between Kis and AcerKis neurons. The time to peak current was measured as the delay between the onset of the pulse of ACh and the maximal current. This value was significantly faster in AcerKis neurons (1.1 ± 0.1 s, *n* = 5) when compared to Kis neurons (1.7 ± 0.1 s, *n* = 10) (Fig. 2e). In addition, ACh-induced charge entry (calculated from the integral of the current according to the Eq. (3)) and current density were also determined in both Kis and AcerKis neurons. For that purpose, the mean values of the surface areas were calculated from the estimated neuron cell body diameters illustrated in Fig. 2f. In this case, the soma of Kis neurons were usually 9.0 ± 0.3 µm (*n* = 56) in diameter (mean neuron surface area of 271.8 ± 17.7 µm^2^) and 8.8 ± 0.2 µm (*n* = 57) in diameter (mean neuron surface area of 258.3 ± 15 µm^2^) for AcerKis neurons. This analysis showed a clear increase in ACh-induced charge entry in AcerKis neurons (25.1 ± 19.3pC; *n* = 5) compared to Kis neurons (2.5 ± 1.2pC; *n* = 4) (Fig. 2g). A similar increase in ACh current density was also observed in AcerKis neurons (0.10 ± 0.07pC/µm^2^; *n* = 5) when compared to Kis neurons (0.01 ± 0.005 pC/µm^2^; *n* = 4) (Fig. 2h). In addition, it has been proposed that the protein content of plasma membrane can influence membrane capacitance (Cm) by affecting the dielectric properties of the membrane. Therefore, the whole-cell patch-clamp technique in voltage-clamp mode was applied to monitor changes in Cm. In this case, a simplified circuit can be used (Fig. 3b) assuming that the neuronal membrane is isopotential, and that no voltage-dependent conductances are active. This model circuit predicts that the current transient, following a hyperpolarized voltage pulse, will have an exponential time course (Fig. 3a, c). Kis and AcerKis neurons were clamped at −50mV, pulses of −10mV (15 ms in duration) were applied, and average capacitive transients were obtained from Kis and AcerKis neurons (Fig. 3a, c). The decay phase of the transient was well-fitted with a single exponential (ז=0.062 ± 0.002 ms, correlation coefficient *r*^2^ = 0.970; *n* = 10, Fig. 3a, d), indicating that the simplified Cm/Rm circuit (i.e., single compartment model, Fig. 3b) provided an adequate description of Kis neurons. Interestingly, the decay phase of the transient in AcerKis neurons was best described by the sum of two exponentials giving the corresponding slow (τs) and fast (τf) time constants (τs = 0.040 ± 0.002 ms, τf = 0.020 ± 0.001 ms, respectively, *r*^2^= 0.970, *n* = 9; Fig. 3c, d). These results obtained for Kis and AcerKis neurons suggest that variation in nAChRs density could affect Cm in AcerKis neurons. All together, these data indicate that the relationship found between, faster time to peak, increased charge entry and ACh current density, irrespective of the average soma diameter (Fig. 2f) and changes in Cm, may be explained by higher density of functional nAChRs expressed in AcerKis neurons. This contributes notably to increase the flow of ions through nAChRs, which thereby produce larger ACh-induced current amplitude in AcerKis than in Kis neurons.

### Functional expression of different types of nAChRs in neurons isolated from AcerKis strains

**Fig. 4:**
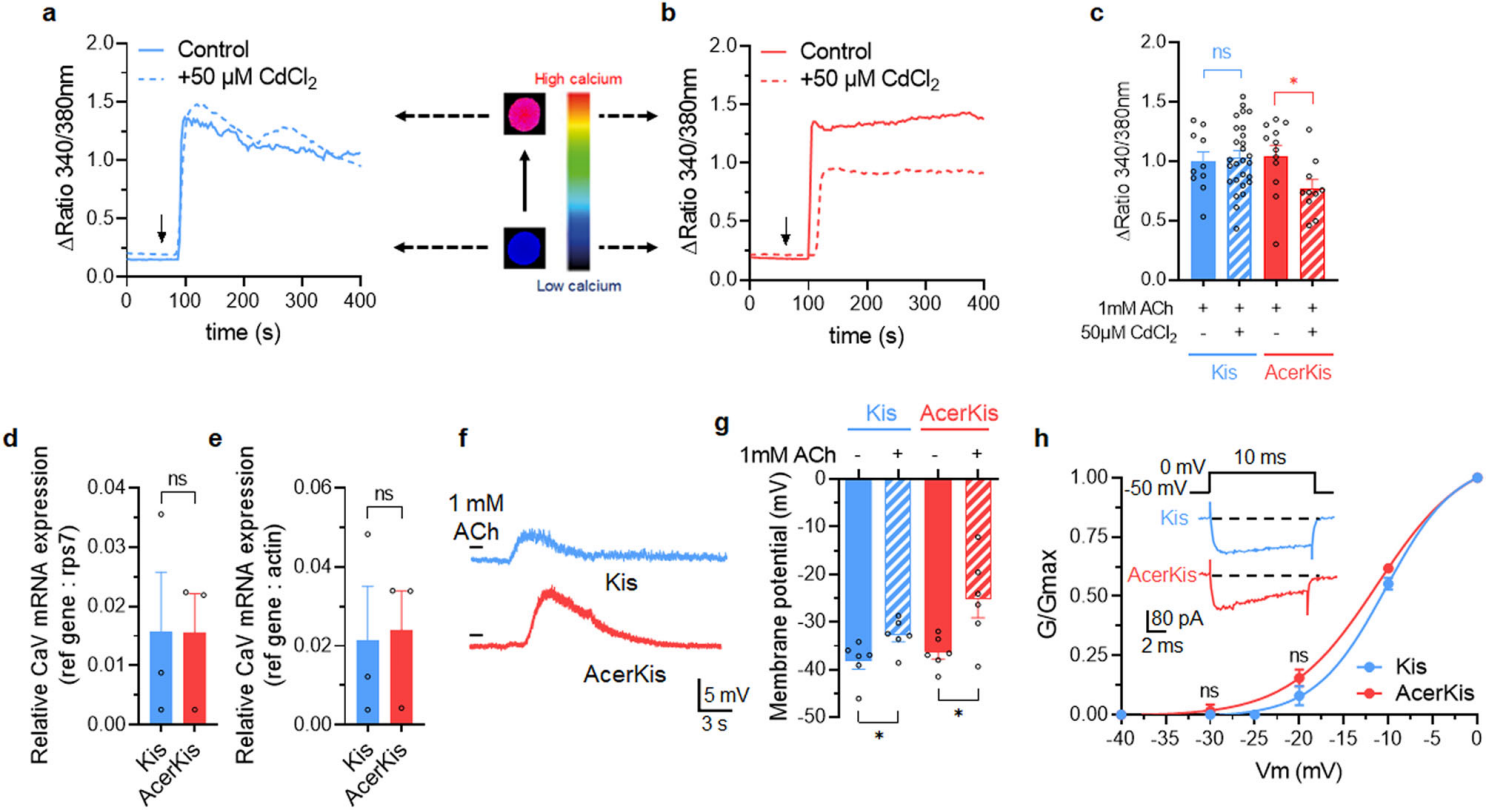
Acetylcholine activates voltage-dependent calcium channels in AcerKis neurons. **a**–**c** Bath application of acetylcholine (ACh, 1 mM) increased intracellular free calcium concentration in Fura-2-loaded Kis (**a**) and AcerKis (**b**) neurons. Pretreatment with the voltage-gated calcium channel blocker CdCl_2_, (50 µM) significantly reduced ACh-induced [Ca^2+^]_i_ rise in AcerKis neurons (**b, c**) but did not produce any effect on [Ca^2+^]_i_ elevation in Kis neurons (**a**, **c**). *Inset* shows images of Fura-2 fluorescence of a single neuron cell body isolated from Kis and AcerKis strains after application of 1 mM ACh. Bars represent mean ± S.E.M. (*n* = 10–27 for Kis neurons and *n* = 10–12 for AcerKis neurons). The statistical test used was Student unpaired *t*-test, *p < 0.05; ns, non-significant. **d**, **e** Comparative histograms illustrating the relative voltage-gated calcium channel mRNA expression in Kis and AcerKis strains. Note that there is no significant difference in the relative mRNA expression of calcium channels between Kis and AcerKis strains. Bars represent mean ± S.E.M. (*n* = 4). The statistical analysis was made by using Analysis of Variance (one-way ANOVA), ns, non-significant. **f** Effects of ACh on Kis and AcerKis neuronal nAChRs studied under current-clamp condition. Pulse application of ACh (1 mM, 3 s in duration) produced a membrane depolarization in AcerKis neurons greater than in Kis neurons. **g** Histogram illustrating the effects of the ACh-induced membrane depolarization in Kis and AcerKis neurons. Bars represent mean ± S.E.M. (*n* = 6), The statistical test used was Student unpaired *t-*test, *p < 0.05. **h** Characterization of the voltage-gated calcium current studied under voltage-clamp condition according to the protocol indicated above each current trace. *Inset* represents typical examples of the voltage-dependent inward calcium current recorded in Kis and AcerKis neurons. Currents are capacity- and leak-corrected. Voltage dependence of the normalized conductance of the inward calcium current, in Kis (*n* = 3–5) and AcerKis (*n* = 3–7) neurons, calculated according to Eq. 6. Bars represent mean ± S.E.M. The statistical test used was Student unpaired *t*-test, ns, non-significant. In all cases, the number of experiments (*n*) are biologically independent samples.

**Fig. 5:**
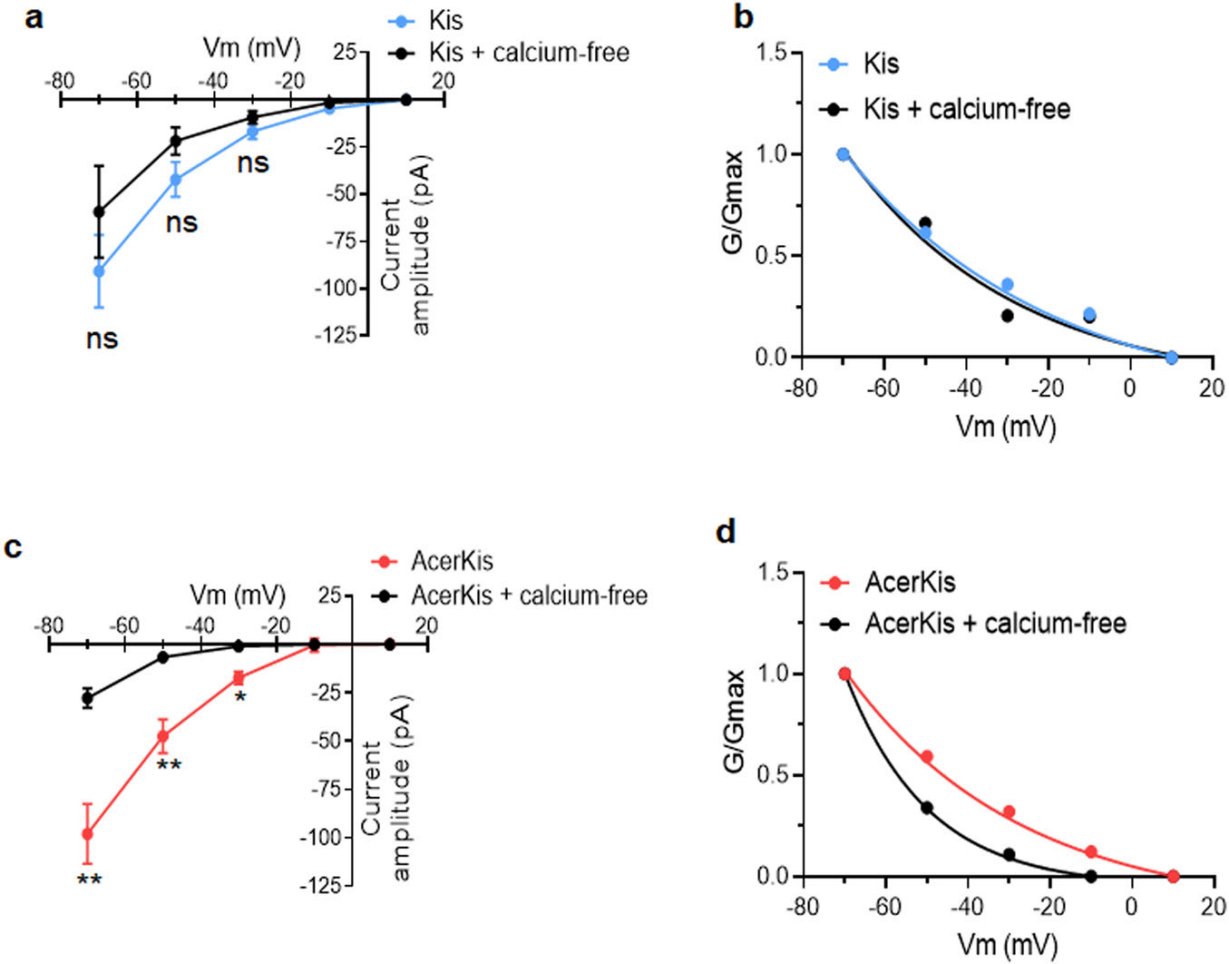
Pretreatment with EGTA-buffered calcium-free solution reduces the ACh-induced current amplitude in AcerKis neurons but has no effect in Kis neurons. **a**, **c** The ACh-evoked inward current currents (pulse application of 1 mM ACh, 3 s in duration) were recorded for holding potentials ranging from −70 to +10 mV with 10 mV steps every 2 min in control and in the presence of EGTA-buffered calcium-free solution in Kis neurons (**a**) and in AcerKis neurons (**c**). The mean values of the peak current amplitude recorded in Kis neurons (*n* = 6–9) and in AcerKis neurons (*n* = 3–9) were plotted as a function of the holding potential. Bars represent mean ± S.E.M. The statistical test used was Student unpaired *t*-test, **p < 0.01; *p < 0.05; ns, non-significant. The number of experiments (*n*) are biologically independent samples. It should be noted that the ACh-induced current amplitude recorded in AcerKis neurons is significantly reduced in the presence of EGTA-buffered calcium-free solution. **b**, **d** Normalized chord conductances of ACh-induced current (G/Gmax) are plotted versus membrane potential in control and in the presence of EGTA-buffered calcium-free solution in Kis (**b**) and AcerKis (**d**) neurons. Changes in the current amplitude and the associated reduction in the maximal calculated conductance indicate that nAChRs activated by ACh (1 mM) are at least permeable to calcium in AcerKis neurons.

It is known that both extracellular and intracellular calcium modulate the agonist-induced currents of native insect neuronal nAChRs^28,32–34^. Because α-bgt tested did not appear to discriminate between different types of nAChRs expressed in AcerKis neurons (see Fig. 1h), we found an alternative way of separating nAChRs by studying the putative influence of extracellular calcium on ACh-evoked response in Kis and AcerKis neurons. Using calcium imaging, comparative experiments were performed in isolated Fura-2 loaded Kis and AcerKis neurons. As shown in Fig. 4a, b, ACh (1 mM) produced an elevation in [Ca^2+^]_i_, followed by a sustained elevated level in both Kis and AcerKis neurons. We next examined the source of the intracellular calcium rise observed in both neurons. When the experiments were performed in the presence of CdCl_2_ (50 µM), a voltage-gated calcium channel blocker, the ACh-induced elevation in [Ca^2+^]_i_ was significantly reduced (by 26%) in AcerKis neurons whereas CdCl_2_ had no effect on the intracellular calcium rise in Kis neurons (Fig. 4c). These results suggest that the elevation in [Ca^2+^]_i_ in AcerKis neurons results, in part, from extracellular calcium through plasma membrane calcium channels. By contrast, voltage-gated calcium channels seemed not to be involved in Kis neurons. One possible explanation of this contrasting result may be the difference of the expression level of voltage-gated calcium channels between Kis and AcerKis neurons. Using transcriptional analysis by RT-qPCR carried out on both mosquito strains, we demonstrated that no differences were found in the α1-subunit of voltage-gated calcium channel mRNA levels between Kis and AcerKis neurons (Fig. 4d, e). Consequently, these results raise the question whether the voltage-gated calcium channels can differently be activated by ACh in both Kis and AcerKis neurons. To address this, we performed current-clamp experiments to compare the ACh-induced membrane depolarization in Kis and AcerKis neurons. The membrane depolarization response following pulse application of ACh (1 mM, 3 s in duration) was significantly greater in AcerKis than in Kis neurons (Fig. 4f, g, *n* = 6). To further demonstrate that ACh-mediated response was sufficient to depolarize AcerKis neurons to voltage-gated calcium channel threshold, we next focused on the study of activation properties of voltage-gated calcium current. The solutions used to record whole-cell calcium currents were designed to eliminate interferences from outward potassium and inward sodium currents (see methods). The electrophysiological protocol used to record inward calcium currents was based on previous findings indicating the existence of High Voltage-Activated (HVA) calcium channels (CaV1) in *Anopheles gambiae* neurons^27^. As shown in Fig. 4h*(inset*), inward calcium currents in both Kis and AcerKis neurons were elicited by a voltage step from −50 mV to 0 mV (10 ms in duration). The total inward current activated at these potentials did not inactivate completely within the duration of the test pulse. The mean amplitudes of the calcium currents were −85.90 ± 16.83pA and −86.14 ± 10.28 pA in Kis and AcerKis neurons, respectively (*n* = 10). It should be noted that bath application of 50 µM CdCl_2_ completely blocked the inward calcium current in both neurons. The voltage range for activation of the inward calcium current can be assayed from the amplitude of the peak currents after pulses of various amplitudes (from −40 mV to 0 mV, in 10 mV increments, holding potential of −50 mV). Figure 4h illustrated normalized plots of the conductances as a function of the voltage activation. To compare more quantitatively the voltage dependency of the two conductances, the voltage relationships were fitted by a Boltzmann Eq. (7). As shown in Fig. 4h, there was no difference in the voltage dependence of calcium conductances between Kis and AcerKis neurons (*n* = 3–7). Both inward calcium currents were activated in a positive potential range around −25mV. By comparing the results from Kis neurons (Fig. 4f, g), the ACh-induced depolarization recorded in AcerKis neurons is strong enough to reach the voltage-gated calcium channel activation threshold and to generate calcium influx. This confirms the effect of CdCl_2_ on the [Ca^2+^]_i_ rise that is only observed in AcerKis and not in Kis neurons (Fig. 4a, b). It also demonstrates a clear difference in the pattern of the nAChRs density in AcerKis neurons, when compared to Kis neurons. They can elevate intracellular calcium levels indirectly because of their ability to further depolarize the neuronal cell body membrane, which thereby activate cell body voltage-gated calcium channels previously described^35–37^. It is also known from the genome sequence that *Anopheles gambiae* can express multiple classes of nAChRs^38–40^. Some of them are capable of elevating [Ca^2+^]_i_ directly because of their permeability to calcium. Because CdCl_2_ failed to completely block the intracellular calcium rise observed in Kis and AcerKis neurons (Fig. 4a, b), experiments were performed in an EGTA-buffered calcium free superfusing solution (Supplementary Fig. 1). Using calcium imaging, pulse application of ACh (1 mM, 3 s in duration) was ineffective in producing any [Ca^2+^]_i_ rise in both Kis and AcerKis neurons (Supplementary Fig. 1a, e). Interestingly, the whole-cell patch clamp studies indicated that the concentration-dependent increase of ACh-induced inward current amplitudes were not affected in Kis neurons pretreated with calcium free extracellular solution (Supplementary Fig. 1b–d). By contrast, ACh-evoked inward current amplitude was significantly reduced in AcerKis neurons but only for concentrations above 100 µM (Supplementary Fig. 1f–h). These results suggest that nAChRs expressed in Kis neurons should not be permeable to calcium ions. On the contrary, the ACh-induced current amplitude reduced by removal of calcium ions from the extracellular solution indicates that the inward current in AcerKis neurons is carried at least in part by calcium. This last effect only observed for higher concentration than 100 µM, supports the idea that distinct types of nAChRs are present on the AcerKis neurons, which thereby can also be distinguished on the basis of their differential sensitivity to ACh. Finally, evaluation of nAChRs calcium permeability in AcerKis neurons compared to Kis neurons was further studied. ACh-induced currents (pulse application of 1 mM ACh, 3 s in duration) were recorded for steady-state holding potentials ranging from −70 to +10 mV (in 10-mV increments) in the control condition and after pretreatment with the calcium-free extracellular solution (Fig. 5a, c, *n* = 3-9). It should be noted that using this protocol, voltage-gated calcium channels are inactivated. In both cases, ACh failed to evoke detectable current for potentials above 0 mV, indicating a strong rectification of the nAChRs. The calcium-free solution induced no significant reduction of neither the ACh-evoked current amplitude nor the chord conductance well described by a monoexponential fit, through nAChRs in Kis neurons (Fig. 5a, b). When AcerKis neurons were pretreated with calcium-free extracellular solution, the amplitude of the ACh-induced current was very weak and significantly reduced compared to control condition (Fig. 5c). Changes in the current amplitude and the associated reduction in the maximal calculated conductance (Fig. 5d), confirm that nAChRs, activated in the concentration of ACh ranging from 100 µM to 1 mM (Supplementary Fig. 1f, h), are at least permeable to calcium in contrast to other nAChRs activated for the lower concentration range (Supplementary Fig. 1f, g). Regarding the [Ca^2+^]_i_ rise recorded in Kis neurons, many other direct and/or indirect mechanisms can rapidly increase intracellular calcium levels. This will need further investigations, which are not the topic of the present study.

### Acetylcholine has different effects on nAChRs in neurons isolated from *Anopheles gambiae* KdrKis strains

**Fig. 6:**
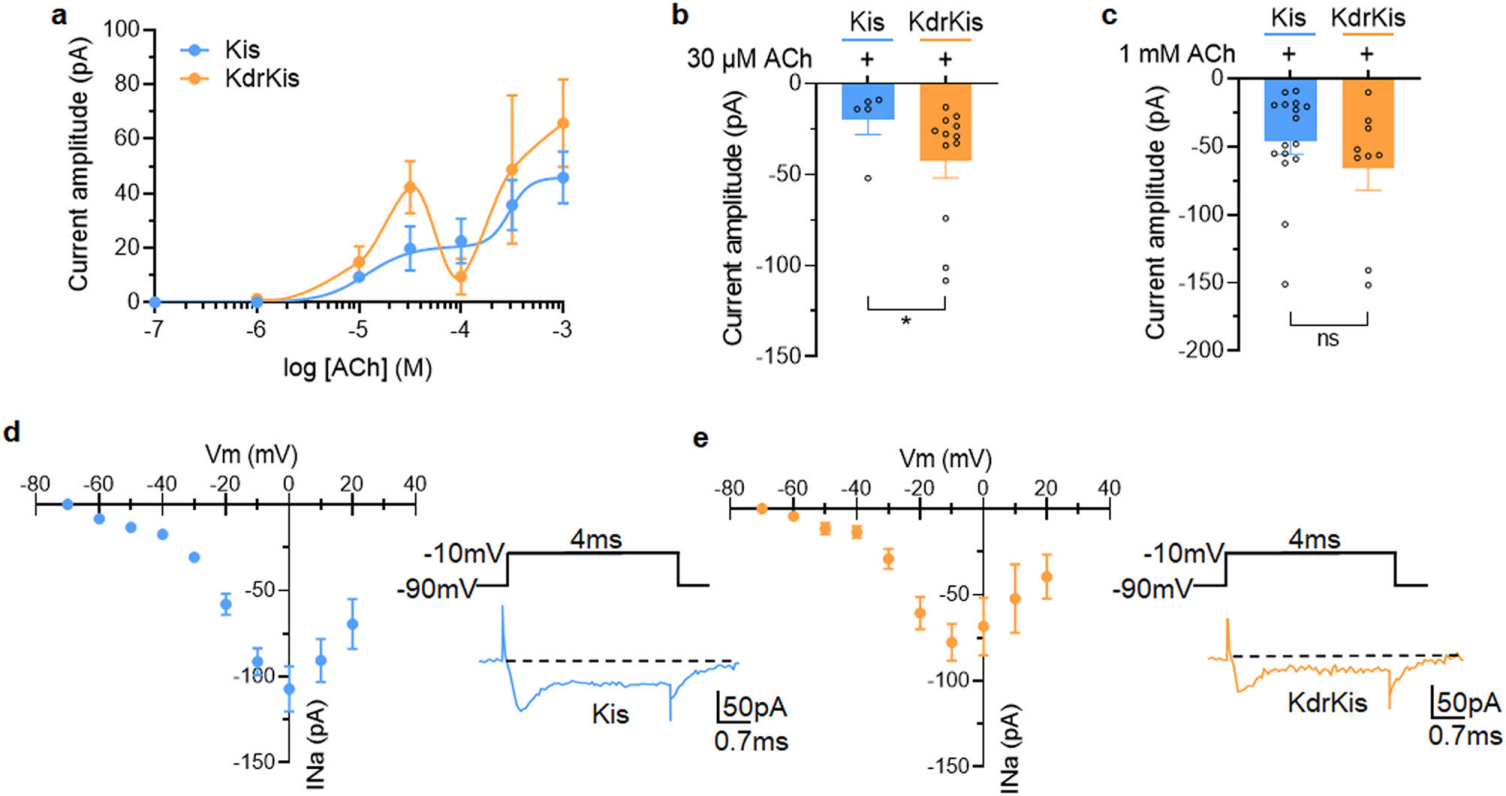
Acetylcholine has different effects on nAChRs in neurons isolated from *Anopheles gambiae* KdrKis strains. **a** Superimposed semilogarithmic dose-response curves for the ACh-induced currents recorded at a holding potential of −50mV in isolated neurons from mosquito strains Kis and KdrKis, as indicated on the graph. Note that for concentration lower than 100 µM (i.e., 30 µM), ACh produced higher current amplitude than in Kis neurons. Data are mean ± S.E.M. (*n* = 3–16). **b**, **c** Comparative histogram showing the effects of pulse application of ACh (3 s in duration) tested at 30 µM (**b**, *n* = 5 and *n* = 12, for Kis and KdrKis, respectively) and 1 mM (**c**, *n* = 16 and *n* = 9 for Kis and KdrKis, respectively). Bars represent mean ± S.E.M. Statistical test used was the Mann–Whitney test, *p < 0.05; ns, non-significant. **d**, **e** Voltage dependence of the inward sodium current recorded in Kis and KdrKis neurons. Current–voltage relationship constructed from values of maximum current amplitude plotted as a function of test potentials (holding potential −90 mV) in Kis (**d**, *n* = 6–7) and KdrKis (**e**, *n* = 4–7). *Insets* represent typical examples of inward sodium currents elicited with a 4 ms depolarizing pulse to −10 mV applied from a holding potential of −90 mV. Currents are capacity- and leak-corrected. Note that in both Kis and KdrKis neurons, the inward sodium current present an incomplete inactivation during the maintained depolarization. However, the current amplitude is smaller in KdrKis neurons compared to that of Kis neurons. Data are mean ± S.E.M. In all cases, the number of experiments (*n*) are biologically independent samples.

Evaluation and comparison study of ACh were performed on KdrKis neurons isolated from the *Anopheles gambiae* strain, KdrKis (*kdr*, L1014F). Using whole-cell patch clamp technique, we showed that pulse application of ACh induced multiphasic inward current amplitude depending on the concentrations tested (Fig. 6a). The mean values of the ACh-induced current amplitudes, plotted against the logarithm of the non-cumulative concentrations of ACh, revealed an unexpected first component recognized as a « bell-shaped » between 1 µM and 100 µM, with a higher maximum amplitude reached at 30 µM, when compared to Kis neurons (−42.3 ± 9.5 pA, (*n* = 12) and −19.8 ± 8.1 pA, (*n* = 5) in KdrKis and Kis neurons, respectively; Fig. 6a, b). For higher concentrations than 100 µM, inward current amplitude further increased before reaching similar maximum amplitudes obtained at 1 mM, as compared with Kis neurons (Fig. 6a, c).

**Fig. 7:**
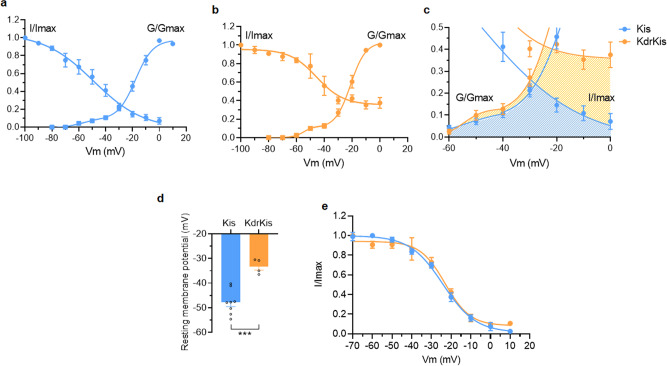
Biophysical properties of the voltage-gated sodium currents in neurons isolated from Kis and KdrKis strains influence membrane potential and voltage-gated calcium channel availability. **a**, **b** Voltage-dependence of steady-state inactivation and conductance parameters of the voltage-gated inward sodium current in Kis (**a**, *n* = 7–8) and KdrKis (**b**, *n* = 4–6) neurons. Data points are mean values ± S.E.M. Smooth curves were fitted through the data points using Boltzmann distributions (see Eqs. 8 and 9 for normalized conductance and inactivation, respectively). **c** Enlarged colored areas indicate overlap between activation and inactivation curves for Kis (blue) and KdrKis (orange) neurons, which is expected to result in a window current; this window current being largest for KdrKis voltage-gated sodium channels. **d** Comparative histogram illustrating the values of the resting membrane potential recorded under current-clamp condition in Kis and KdrKis neurons. Bars represent mean ± S.E.M. (*n* = 8 and *n* = 4 for Kis and KdrKis neurons, respectively). The statistical test used was Student unpaired *t*-test, **p < 0.01. **e** Voltage-dependent steady-state inactivation of the voltage-gated calcium current studied with the two-pulse voltage-clamp protocol (see text for details) in Kis (*n* = 7) and KdrKis (*n* = 4) neurons. Smooth curves of inactivation were fitted through data points using Boltzmann distribution (Eq. 9). Data are mean ± S.E.M. In all cases, the number of experiments (*n*) are biologically independent samples.

**Fig. 8:**
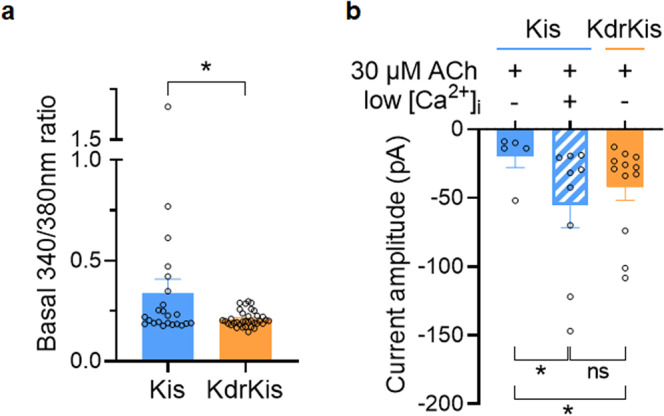
Lowering intracellular calcium concentration in Kis neuron cell body mimicks the ACh-induced current amplitude recorded in KdrKis neurons. **a** Comparative histogram illustrating the steady-state resting intracellular calcium level (expressed as ratio 340/380 nm) measured in Fura-2-loaded Kis (*n* = 11) and KdrKis (*n* = 36) neuron cell bodies. Bars represent mean ± S.E.M. Statistical test used was the Mann–Whitney test, *p < 0.05. **b** Comparative histogram illustrating the effect of the internal pipette solution containing low intracellular calcium concentration (0.01 µM) on the ACh-induced current (*n* = 9) amplitude in Kis neurons (*n* = 5) compared to KdrKis (*n* = 9) and Kis neurons in control conditions. Note that lowering intracellular calcium concentration increases the ACh-induced current amplitude in Kis neurons, an amplitude, which is very close to that of recorded in KdrKis neurons in control. Bars represent mean ± S.E.M. Statistical test used was the Mann–Whitney test, *p < 0.05, ns, non-significant. In all cases, the number of experiments (*n*) are biologically independent samples.

To gain further insight into why L1014F mutation increases ACh-evoked current amplitude at relatively low concentration (i.e., 30 µM) in KdrKis neurons, we first investigated under voltage-clamp condition the biophysical properties of the voltage-gated sodium current in both Kis and KdrKis neurons in the presence of potassium and calcium channel blockers (see methods). Figure 6d, e (*insets*) shows typical examples of inward sodium currents elicited by a voltage step from −90 to +10 mV (4 ms in duration) in Kis and KdrKis neurons. It should be noted that the inward sodium current did not inactivate completely within the duration of the test pulses. As previously shown in Kis neurons, the sodium current was completely blocked by TTX^27^. For comparison, Fig. 6d, e illustrates the averaged current-voltage (I/V) relationship for the peak current in Kis and KdrKis neurons. In both cases, the inward sodium current began to appear at a membrane potential above −60 mV, rising steeply and peaking at 0 mV and −10mV, in Kis and KdrKis neurons, respectively. In KdrKis neurons, however, the maximum peak current amplitude was smaller (−77.5 ± 10.7 pA at −10 mV, (*n* = 4–7) than that of recorded in Kis neurons (−107.1 ± 13.2 pA at 0 mV in Kis neurons, *n* = 6–7). The inward sodium current then decreased to an extrapolated potential around +65 mV, a value close to the calculated Nernstian equilibrium potential for sodium ions in our experimental conditions (+73.6 mV). We next focused on the study of activation and inactivation properties of the inward sodium current in Kis and KdrKis neurons (Fig. 7a, b). The voltage range for activation of the inward sodium currents can be assayed from peak current amplitudes after pulses of various amplitudes (−80 to +10 mV, in 10-mV increments), from a holding potential of −90 mV. Conductance values were calculated by dividing the peak current amplitude by the driving force at each potential and normalizing to the maximum conductance according to the Eq. (6). As illustrated (Fig. 7a, b), the voltage curves of the conductance revealing a biphasic aspect were best fitted according to a double Boltzmann Eq. (8). The corresponding activation parameters were measured for Kis and KdrKis neurons and summarized in the Supplementary Table 1. To measure the voltage-dependence of the inward sodium current steady-state inactivation, a two-pulse voltage-clamp protocol was used. Inactivation properties were studied by applying a conditioning pulse between −110 and +20 mV (10-mV increments) that was long enough (500-ms) to allow the inactivation process to reach its steady-state level. Thereafter, the membrane potential was stepped back to the holding potential (−90mV) for 1 ms to ensure that the activation variable remained constant, and a 15 ms test pulse was applied to −10mV. The inactivation curves were obtained by plotting the amplitude of the peak sodium currents against the conditioning potential (Fig. 7a, b). The smooth line corresponds to the best fit through the mean data points using a single Boltzmann distribution (see Eq. 9). The values of the voltage-dependence of steady-state inactivation parameters were given in Supplementary Table 1 for Kis and KdrKis neurons. The amplitude of the peak inward sodium current decreased as the depolarizing conditioning pulse was made more positive, indicating that fewer channels were available for activation at positive potentials (Fig. 7a, b). Although the voltage dependence of inactivation was not different between Kis and KdrKis sodium channels (Supplementary Table 1), the inactivation was incomplete in KdrKis neurons (Fig. 7b) when compared to Kis neurons (Fig. 7a). The steady-state inactivation and conductance voltage (activation) curves for Kis sodium currents intersected near –30 mV, while those for KdrKis intersected around –25 mV (Fig. 7c). The resulting increase in overlap of activation and inactivation of KdrKis sodium channels predicts a larger steady-state window current, which is expected to generate a «steady» influx of sodium into the neurons and a resulting tonic depolarization of KdrKis neurons (Fig. 7c). Therefore, current-clamp experiments were performed to measure the resting membrane potential in Kis and KdrKis neurons. As presented in Fig. 7d, we confirmed that the resting membrane potential was significantly lower in KdrKis neurons (−33.2 ± 1.7 mV, *n* = 4), compared to Kis neurons (−47.8 ± 1.9 mV, *n* = 8). It is known that the calcium influx is an important determinant of the regulation of nAChR sensitivity to ACh, suggesting that calcium is of special interest because of its implications for nAChR function. We previously indicated that nAChRs expressed in KdrKis differ in their sensitivity to low concentration ACh (i.e., 30 µM) compared to those present in Kis neurons (Fig. 6a). Furthermore, the fast inactivation of voltage-dependent calcium channels is an important, intrinsic regulatory mechanism that helps to control calcium entry into neurons. We then studied the putative consequence of the depolarized membrane potential on the contribution of calcium channel activated in KdrKis neurons. For that purpose, inactivation properties were studied by applying a 200-ms conditioning pulse between −70 and +10 mV (in 10-mV increments). Thereafter, the membrane potential is stepped back to the holding potential (−50 mV) for 5 ms before a 10-ms test potential to 0 mV. The peak current at different holding potentials was normalized to the maximum peak current recorded from the holding potential of −70mV. As shown in Fig. 7e, the amplitude of the peak calcium current decreased as the depolarizing conditioning pulse was made more positive in both Kis and KdrKis neurons. It is interesting to note that in KdrKis neurons, at resting membrane potential (−33.2 ± 1.7 mV), only 80% of calcium channels were in an activable state, compared to Kis neurons (100% at −47.8 ± 1.9 mV, Fig. 7d, e). In parallel and based on these results, using calcium imaging, we compared the steady-state resting intracellular calcium level in Fura-2 loaded Kis and KdrKis neurons (Fig. 8a). Interestingly, a significant decrease was observed in resting calcium level in KdrKis neurons compared to Kis neurons. Taken together, these results prompt us to examine whether the effect of reduced [Ca^2+^]_i_ could also modify the nAChR sensitivity to 30 µM ACh in Kis neurons, compared to KdrKis neurons. When relatively low calcium concentration (i.e., 0.01 µM rather than 0.1 µM, see methods) was introduced into the Kis neuron through the patch pipette, the ACh-induced current had a very similar amplitude compared to that of in KdrKis neurons (Fig. 8b). These results indicate that low [Ca^2+^]_i_ was responsible for the increase in ACh-induced current amplitude, observed at relatively low concentration of ACh, in KdrKis neurons (Figs. 6a and 8b).

### The effectiveness of the neonicotinoid insecticide, clothianidin differs with changes in neuronal nAChR properties following the mutations G119S in AcerKis strain and L1014F in KdrKis strain

**Fig. 9:**
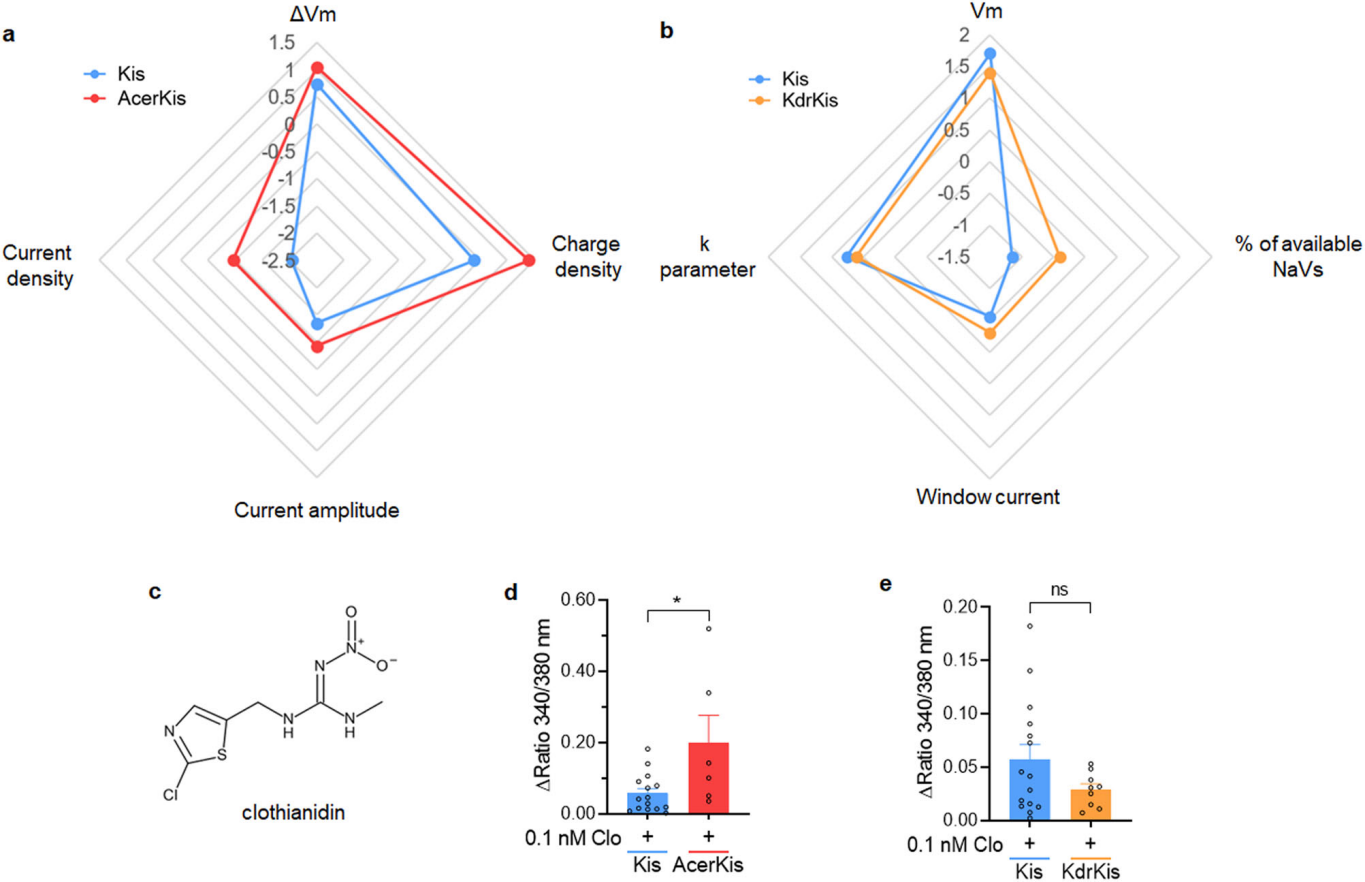
Summary of the physiological parameters changes observed in neurons isolated from AcerKis and KdrKis strains and neonicotinoid insecticide clothianidin effectiveness in increasing neuronal [Ca^2+^]_i_. **a**, **b** Radargraph comparing the changes (logarithmic scale) in the most important physiological parameters observed in Kis, AcerKis (**a**) and KdrKis (**b**) neurons. The effects of clothianidin (**c**) was studied in Fura2-loaded Kis, AcerKis and KdrKis neuron, using calcium imaging. **d**, **e** Comparative histogram illustrating the effects of clothianidin (0.1 nM) on the [Ca^2+^]_i_ recorded in AcerKis (**d**) and KdrKis (**e**) neurons compared to Kis neurons. Note that the [Ca^2+^]_i_ rise was greater in AcerKis than in KdrKis, indicating the key role of intracellular calcium level in the modulation of the nAChR sensitivity to clothianidin. Bars represent mean ± S.E.M. (*n* = 15, *n* = 6 and *n* = 9 for Kis, AcerKis and KdrKis neurons, respectively). The statistical test used was Student unpaired *t*-test, *p < 0.05, ns, non-significant. The number of experiments (*n*) are biologically independent samples.

Changes in the neuronal physiological properties including nAChR functions observed in AcerKis and KdrKis neurons compared to Kis neurons are given in the radargraph illustrated in Fig. 9a, b. Briefly, 4 specific parameters were clearly differentially affected in Kis, AcerKis and KdrKis neurons. We then studied in both in vitro and in vivo if these physiological modifications can be associated with an alteration in insecticide effectiveness. Comparative experiments were performed with the neonicotinoid insecticide, clothianidin, a new class of chemistry for public health, known to act as an agonist on insect nAChRs^41–44^. Using calcium imaging, clothianidin (0.1 nM, Fig. 9c) was tested in Fura-2 loaded neurons isolated form Kis, AcerKis and KdrKis strains (Fig. 9d, e). Clothianidin induced a significant intracellular calcium rise in AcerKis neurons in contrast to the lack of effect of the insecticide on [Ca^2+^]_i_ in KdrKis neurons compared to Kis neurons.

To verify that compensation mechanisms also influence insecticide efficacy in vivo, clothianidin was tested on Kis, AcerKis and KdrKis larvae. In each strain, between 2400 to 3200 larvae of the late-third to early-fourth instar (700 subject larvae and 100 control larvae in one test) were tested for their susceptibility to clothianidin after 24 and 48 h exposure. The calculated averaged lethal concentrations LC_50_ and LC_95_ are summarized in Table 1 with their 95% confidence interval (CI). After 24 h exposure to clothianidin, LC_50_ and LC_95_ were similar among the three strains as they ranged from 0.018 to 0.032 mg/L, and from 0.042 to 0.081 mg/L, respectively. After 48 h exposure to clothianidin, LC_50_ and LC_95_ were shifted towards lower doses as they ranged from 0.012 to 0.029 mg/L, and from 0.024 to 0.059 mg/L, respectively. This global range shift was assimilated to a longer exposition to insecticide. Interestingly, with a 48 h contact time, LC_50_ was lower for AcerKis strain and higher for KdrKis strain when compared with Kis strain. These results demonstrate that clothianidin has a better efficacy on AcerKis larvae, and a lower efficacy on KdrKis larvae when compared with the reference Kis strain. They are in line with our in vitro data, therefore confirming that cellular and molecular compensation mechanisms resulting from G119S and L1014F mutations modulate the response to insecticides targeting nAChRs efficacy in vivo.

**Table:** Larval susceptibility of *Anopheles gambiae* Kis, AcerKis and KdrKis to clothianidin.

Mosquito strain	*n*^a^	Contact time	LC_50_ (95% CI)	LC_95_ (95% CI)
Kis	4	24 h	0.030 (0.026–0.036)	0.062 (0.05–0.101)
	4	48 h	0.019 (0.015–0.022)	0.039 (0.032–0.057)
AcerKis	4	24 h	0.018 (0.014–0.026)	0.042 (0.031–0.076)
	3	48 h	0.012 (0.010–0.013)	0.024 (0.021–0.034)
KdrKis	4	24 h	0.032 (0.024–0.041)	0.081 (0.059–0.13)
	3	48 h	0.029 (0.025–0.033)	0.059 (0.049–0.078)

^a^One test includes 700 subject larvae and 100 control larvae. The number of experiments (*n*) are biologically independent samples. Lethal concentrations (LC) and confidence intervals (CI) are expressed in mg/L.

## Discussion

Our results show that compensatory mechanisms with no previous known functions, play a key role in conferring changes in insecticide efficacy in two strains of resistant *Anopheles gambiae* AcerKis and KdrKis through an alteration of the cholinergic system involving nAChRs.

These compensatory mechanisms appear to be the consequences of the point mutation in the voltage-gated sodium channels^5,9,24^ and AChE1 genes^25,26,45^ characterized in KdrKis and AcerKis *Anopheles gambiae*, respectively. In the AcerKis strain, previous findings and this study reveal that AChE activity is strongly reduced. It has been demonstrated that the 119 position is close to the catalytic Serine (S200). The G-to-S substitution reduces accessibility to substrate, such as ACh by steric hindrance^30,31,45,46^. In the insect central nervous system (CNS), the evoked release of ACh activates extrasynaptic and/or synaptic homomeric/heteromeric nAChRs^47–49^. ACh action is terminated by AChE. The first effect of the reduced AChE enzymatic activity has been described at synaptic level. Previous findings have reported that any means that increased ACh concentration within the synaptic cleft can induce direct activation of presynaptic mAChRs (M2 mAChR-subtypes) involved in the negative feedback mechanism, decreasing subsequent release of ACh^48^. Beside this effect, the increased concentration of ACh exposure can desensitize/inactivate nAChRs^50,51^, which will alter neuronal function. Although in AcerKis strains, a potential advantage can be gained *via* the G119S mutation conferring resistance to insecticides, there is an opposing need to conserve normal physiological function. Because a decreased AChE activity may results in an elevation of non-hydrolyzed ACh concentration, we suggest that the increase in nAChRs density observed in AcerKis neurons (Fig. 9a) is a key adaptation to gradually increasing ACh levels to avoid nAChR desensitization in order to guarantee the normal neuronal physiological function. In other words, a higher nAChRs density will lead to less severe receptor desensitization to compensate prolonged high concentration of ACh exposure, which is expected to be observed with the same high level of ACh in Kis neurons. This indicates that for a given concentration of ACh, « proportional » occupancy increases with increased receptors density. This interesting concept has recently been proposed for human 5-HT3A expressed in *Xenopus* oocytes^52^. Another second mechanism can also be proposed to reinforce the role of this compensatory mechanism. The additional nAChR population permeable to calcium activated by relatively high concentration could also promote recovery of desensitization observed in response during sustained ACh application. In this case, the rise in intracellular calcium concentration can regulate desensitization by altering the balance of kinase/phosphatase function with an increase in cellular phosphorylation promoting recovery of desensitization^36,50,53,54^. This will represent an additional major mechanism for the upregulation of nAChR to avoid desensitization resulting from the high level of ACh present in AcerKis. In this case, the compensatory mechanism could help to reduce the otherwise deleterious effects caused by G119S mutation. Because there is no data available yet, understanding these adaptive mechanisms is expected to provide insight on protection strategies that may be effective against the actions of increased ACh concentration.

Another well-known resistance mechanism related to the point mutation in the insecticide target site in *Anopheles gambiae* is the pyrethroid-resistance associated L1014F mutation in domain II-S6 of the voltage-gated sodium channel (VGSC)^4,5,55–57^. Although the molecular structure of insect and particularly mosquito VGSCs is now characterized^5,56,58^, comparative electrophysiological studies of wild type and modified channels have only been performed with VGSCs functionally expressed in *Xenopus* oocytes^56,59–62^. In this case, modified VGSCs have been obtained either by introducing into the *para* gene construct the mutation conferring substitutions at the L1014 residue or by expressing, for instance, a recombinant mosquito *Aedes aegypti* α*-kdr* mutant, constructed by site-directed mutagenesis^63^. To date there is no data available on the electrophysiological properties of VGSC expressed in neurons isolated from the Kis and KdrKis strains. One of the most interesting feature of the biophysical properties of the sodium current, summarized in Supplementary Table 1, is the expanded range of membrane potentials at which sodium channels will be conducting (i.e., window current). This has never been observed with modified sodium channels (i.e., L1014F) expressed in *Xenopus* oocytes^62^. The resulting large steady-state window current (see Fig. 7c), is expected to depolarize KdrKis neurons. As demonstrated in our study, the physiological impact of this steady-state depolarization is a reduced availability of voltage-gated calcium channels. Although calcium channels have already been described in insect neurons with distinguished physiological functions in the regulation of neuronal excitability and cellular signaling^35,64^, this is the first characterization of functional voltage-gated calcium channels in mosquito neurons with a potential key role in mediating adaptive response, shown as a result of the *kdr* mutation. The critical role of voltage-gated calcium channels in the ACh release and in the calcium signals that contribute to the rapid ACh release is now well established^65^. From our findings, we can hypothesize that the fraction of available calcium channels, reduced by depolarization at neuronal level (Fig. 7d, e), may decrease the amount of ACh released, in vivo, suggesting that relatively low concentrations of ACh will reach nAChRs. This could be compensated by increasing nAChR sensitivity as it is observed in KdrKis neurons since the same low concentration of ACh produces a greater effect than in Kis neurons. Today, the question, which is still open concerns the mechanisms involved to convert low-sensitivity nAChR site into a high-sensitivity nAChR site, allowing nAChRs to exert their optimal action on physiological functions. Previous findings have demonstrated differences in agonist sensitivity of neuronal nAChRs, expressed in various insects^40,66^, which depend on intracellular and/or extracellular calcium^28,32,67^. Our results support the hypothesis that intracellular calcium influences the sensitivity of the nAChRs to ACh in KdrKis neurons (Fig. 8). The ability of ACh to produce stronger effect on nAChR in KdrKis neurons seems to be correlated with the lower steady-state resting calcium level compared to Kis neurons. This is confirmed by using the strategy of lowering intracellular calcium concentration, which increases the ACh-induced current amplitude in Kis neurons. Furthermore, the reduced availability of calcium channels, which thereby decreases calcium influx, reinforces our hypothesis that low intracellular calcium concentration can enhance nAChR sensitivity to ACh in KdrKis neurons. Even if the precise intracellular mechanisms involved remains to be elucidated, previous studies have demonstrated the influence of calcium-dependent intracellular signaling pathways mediating phosphorylation/dephosphorylation in the regulation of insect neuronal nAChR sensitivity to cholinergic agonists^67^.

We also report that these unusual specific calcium-sensitive mechanisms illustrating the existence of original compensatory mechanisms to L1014F and G119S mutations in KdrKis and AcerKis neurons, have fundamental consequences on the efficacy of insecticides. As indicated, changes in functional properties of nAChRs together with the influence of intracellular calcium are considered as important clues to understand the opposite effects of the neonicotinoid, clothianidin observed in vitro and in vivo in AcerKis and Kdrkis strains. The key role of intracellular calcium in mediating modulation of the neonicotinoid efficacy, has previously been reported in other insect neuronal preparations^32,67–69^. The higher density of nAChRs permeable to calcium together with the activation of voltage-gated calcium channels undoubtedly contribute to potentiate the effect of clothianidin in AcerKis neurons. By contrast, the mechanisms involved in the maintenance of low intracellular calcium concentration appear to be responsible for reducing clothianidin efficacy in KdrKis neurons. The interesting aspect of this study is that in vitro studies performed in AcerKis and KdrKis neurons correlate well with the clothianidin-induced lethal effects observed in vivo on larvae. After 48 h clothianidin exposure, LC_50_ is lower for AcerKis strain and higher for KdrKis strain when compared with Kis strain. This strongly suggests that these compensatory mechanisms would also be initiated in the whole insect. Therefore, these data confirm the need for understanding these specific physiological compensatory mechanisms associated with the point mutations to adapt control strategies. This is particularly true for clothianidin, which is now recommended as a vector control product against *Anopheles* species and used as clothianidin‑based formulation^41,42^. In this case, it is interesting to mention that the effectiveness of the formulation containing a mixture of deltamethrin/clothianidin against wild pyrethroid-resistant malaria vectors may be explained by previous studies indicating that deltamethrin, used as synergistic agent, can optimize insecticide efficacy *via* intracellular calcium rise in insect^70^. Because mosquito populations and particularly *Anopheles* species reveal heterogeneous profiles ranging from susceptibility to strong resistance, understanding the compensatory mechanisms related to these point mutations should be taken into account to adapt future strategies^71^ and to ensure full vector control, whatever the resistance mechanisms. This knowledges will optimize insecticide-based vector control, essential to prevent clusters of resistant mosquito populations expressing reduced toxicity to a given treatment.

## Methods

### Mosquito strains and rearing

Three *Anopheles gambiae* female mosquito strains (Kis, AcerKis and KdrKis) were used in this study. The used colony named Kisumu (Kis) was the susceptible resistance-free reference strain. The AcerKis strain is homozygous for the G119S mutation and resistant to both organophosphates and carbamates insecticides^72^. The colony, named KdrKis strain, harbors the L1014F homozygote mutation (*kdr*-west allele) in the gene coding for the voltage-gated sodium channel, which confers resistance to pyrethroids and DDT. The colony was obtained by introgression into the susceptible genetic background of the Kisumu colony the *kdr*-west allele obtained from pyrethroid resistant mosquitoes in Kou Valley, Burkina Faso^73^. Insecticide resistance was confirmed by using insecticide bioassays and genotypic verifications^74^. The colony does not carry any metabolic resistance. Eggs were provided by MIVEGEC laboratory (UMR IRD-CNRS-Montpellier University) from Montpellier, France. Mosquitoes were reared in an 80% humidity and 28 °C temperature environment with a 12 h light/dark photo-cycle. After hatching in demineralized water, larvae are fed daily with Tetramin® fish food. Adults are fed daily with a 10% honey solution. Experiments were performed exclusively on emergent mosquitoes from the day.

### Neuron isolation procedure

Mosquitoes were anesthetized at 4 °C and disposed under binocular microscope. Antennae, maxillary palps, labium and antennal pedicels were removed carefully. Heads were excised and placed in mosquito saline containing 130 mM NaCl, 2.5 mM KCl, 5 mM CaCl_2_, 3 mM MgCl_2_, 5 mM HEPES, 50 mM sucrose, 5% fetal bovine serum, 50 μg/mL streptomycin, 50 UI/mL penicillin, pH was adjusted to 7.2 with NaOH. Neuronal cells were dissociated, identified and maintained at 29 °C for 4 h before experiments were carried out^27^.

### Electrophysiological recordings

Steady-state and voltage-gated ionic currents (voltage-clamp mode) were recorded by using the patch-clamp technique in the whole-cell recording configuration. Membrane potential was recorded under current-clamp mode. Signals were recorded with an Axopatch 200 A amplifier (Axon Instruments, Foster City, CA), filtered at 5 kHz using a 4-pole lowpass Bessel filter. Ionic currents were displayed on a computer with software control pClamp (version 10.0; Molecular Devices) connected to a digitizer (DIGIDATA 1322; Molecular Devices). Patch pipettes were pulled from borosilicate glass capillary tubes (GC 150T-10; Clark Electromedical Instruments, Harvard Apparatus) using a Sutter P-97 (Sutter Instruments). Pipettes had resistances ranging from 5 to 7MΩ when filled with internal pipette solution. The liquid junction potential between bath and internal solution was always corrected before the formation of a gigaohm seal (>2 GΩ).

To record steady-state cholinergic agonist-induced ionic currents, cells were voltage-clamped at a steady-state holding potential of −50mV (except when otherwise stated). To study the voltage-gated calcium currents, mosquito neurons were voltage-clamped at a steady-state holding potential of −50 mV and calcium currents were elicited by 10-ms depolarizing test pluses to 0 mV from the holding potential at a frequency of 0.1 Hz to minimize the rundown of calcium currents. Although leak and capacitive currents were compensated electronically at the beginning of each experiment, subtraction of residual capacitive and leakage currents was performed with an on-line P/4 protocol provided by pClamp. In this procedure, currents elicited by four subpulses from the holding potential with an amplitude one-fourth of the main experimental pulse were added together to compute capacitance and leak-subtracted currents. For the voltage-gated sodium currents, isolated neurons were voltage-clamped at a steady-state holding potential of −90 mV and 4-ms depolarizing test pluses were applied from the holding potential at a frequency of 0.5 Hz. Capacitive currents were compensated electronically at the beginning of each experiment, subtraction of residual capacitive and leakage currents was performed with an on-line P/6 protocol provided by pClamp.

### Solutions and drug applications

For steady-state cholinergic agonist-induced ionic currents (voltage-clamp mode) and membrane potential (current-clamp mode), bath solution superfusing the neuronal cell bodies contained 130 mM NaCl, 2.5 mM KCl, 5 mM CaCl_2_, 3 mM MgCl_2_, 5 mM HEPES, pH was adjusted to 7.2 with NaOH. When the experiments were carried out in calcium-free conditions, CaCl_2_ was removed and compensated with MgCl_2_. Patch pipettes were filled with solution containing 130mM K-Gluconate, 10mM K-Fluoride, 10 mM NaCl, 3 mM MgCl_2_, 0.1 mM CaCl_2_, 1.5 mM EGTA, 1 mM ATP-Mg, 10 mM HEPES, pH was adjusted to 7.2 with KOH.

For the voltage-gated calcium currents, the solutions used were designed to eliminate interference from outward potassium currents by the combination of external tetraethylammonium chloride (TEA-Cl) and 4-aminopyridine (4-AP) and by isotonically substituting potassium with cesium in the patch electrode. Inward sodium current was abolished by omitting NaCl (NaCl was replaced with choline chloride)^27^. The extracellular solution superfusing the cells contained 100 mM Choline chloride, 2.5 mM KCl, 5 mM CaCl_2_, 3 mM MgCl_2_, 30 mM TEA-Cl, 10 mM HEPES, 5 mM 4-AP, pH was adjusted to 7.2 with NaOH. Patch pipettes were filled with an internal solution containing 140 mM CsCl, 7 mM NaCl, 3 mM MgCl_2_, 0.1 mM CaCl_2_, 1.5 mM EGTA, 10 mM HEPES, 3 mM ATP-Mg, pH was adjusted to 7.2 with KOH.

For the voltage-gated sodium currents, the solutions used were designed to eliminate interference from both outward potassium and inward calcium currents. Bath solution superfusing the cells contained 130 mM NaCl, 50 mM TEA-Cl, 5 mM KCl, 2 mM MgCl_2_, 0.5 mM CaCl_2_, 0.5 mM CdCl_2_, 5 mM 4-AP, 10 mM HEPES, pH was adjusted to 7.2 with NaOH 1 M. Patch pipettes were filled with an internal solution containing 140 mM CsCl, 7 mM NaCl, 2 mM MgCl_2_, 2 mM EGTA, 10 mM HEPES, 1 mM ATP-Mg, pH was adjusted to 7.2 with CsOH 1M^27^.

Cholinergic agonists (pulse application 3 s in duration, except when otherwise stated) and pharmacological agents (added to the external solutions) used in the different experiments were applied by a gravity perfusion valve controller system (VC–6 M, Harvard apparatus) controlled by pClamp software (flow rate of perfusion 0.5 mL/min). The perfusion tube was placed within 10μm from the isolated neuron cell body. When necessary, some compounds were prepared in DMSO and then diluted in the bath solution to obtain the different concentrations tested. The highest concentration used in the electrophysiological recordings of DMSO was 0.1%. This concentration of solvent was not found to have any effect on the electrophysiological properties of neuron cell body. All compounds were purchased from Sigma Chemicals (L’isle d’Abeau Chesnes, France). Patch-clamp experiments were conducted at room temperature (20–22 °C).

### Electrophysiology data analysis

The equations used to fit the monophasic (Kis neurons, Eq. 1, consisting of a single Hill equation) and biphasic (AcerKis neurons, Eq. 2, constituted by the sum of two Hill equations) concentration-response curves for ACh were:

1 $${\rm{I}}=\frac{{{\rm{I}}}_{{\max }}}{{1+\left(\frac{{{EC}50}_{1}}{x}\right)}^{{nH}1}}$$

2 $${\rm{I}}={I}_{{\max }}/\left(\frac{{\rm{\alpha }}1}{{1+\left(\frac{{{EC}50}_{1}}{x}\right)}^{{nH}1}}+\frac{1-{\rm{\alpha }}1}{{1+\left(\frac{{{EC}50}_{2}}{x}\right)}^{{nH}2}}\right)$$

where *I*_max_ is the maximal current amplitude and *x* is the agonist concentration, EC50_1_ and EC50_2_ are the concentrations of ACh that gives half-maximal response, nH_1_, and nH_2_ are the Hill coefficient and α_1_ is the fraction of receptors that have an affinity described by EC50_1_.

Charge entry was calculated from the integral of the current according to the Eq. (3):

3 $${\rm{CE}}=\frac{1}{{\rm{zF}}}\int {\rm{It}}$$

where CE is the charge entry, z is the ion elementary charge, *F* is the Faraday constant, *I* is the inward current and t is the time.

The whole-cell patch-clamp technique in voltage-clamp mode was applied to monitor changes in Cm. The decay phase of the transient was well-fitted with a single exponential (Eq. 4) or by the sum of two exponentials (Eq. 5):

4 $${Ic}=A0.{e}^{-t/\tau }$$

5 $${Ic}=A1.{e}^{-t/\tau f}+A2.{e}^{-t/\tau s}$$

where Ic is the capacitive current, A0, A1 and A2 are the corresponding relative current amplitude with time constants τ, τf and τs.

The voltage-dependence of the conductances was calculated as a function of the membrane potential according to Eq. (6) and was fitted according to the Boltzmann Eq. (7):

6 $${\rm{G}}=\frac{{\rm{I}}}{({\rm{Vm}}-{\rm{Ex}})}$$

7 $$\frac{{\rm{G}}}{{{\rm{G}}}_{{\rm{max }}}}=\frac{1}{1+{{\rm{e}}}^{({{\rm{V}}}_{1/2}-{\rm{Vm}})/{\rm{k}}}}$$

where ${\rm{I}}$ is the inward current amplitude, Vm is the potential at which the membrane is clamped and Ex is the equilibrium potential for a given ion. For the voltage-gated sodium current, the conductance-voltage relationship was best fitted according to a double Boltzmann Eq. (8):

8 $$\frac{{\rm{G}}}{{{\rm{G}}}_{{\rm{max }}}}=\frac{1}{1+{{\rm{e}}}^{({{\rm{V}}1}_{1/2}-{\rm{Vm}})/{\rm{k}}1}}+\frac{1}{1+{{\rm{e}}}^{({{\rm{V}}2}_{1/2}-{\rm{Vm}})/{\rm{k}}2}}$$

where G is the sodium conductance, *G*_max_ is the maximal conductance from a holding potential of −90 mV, Vm is the membrane potential applied, *V*_1/2_ is the potential at which half the sodium channels are activated and *k* is the slope factor. Conductance-voltage relationships were fit for each individual cell.

The inactivation curve, obtained by plotting the amplitude of the peak current against the conditioning potential was best fitted according to the Boltzmann distribution (9):

9 $$\frac{{\rm{I}}}{{{\rm{I}}}_{{\rm{max }}}}=\frac{1}{1+{{\rm{e}}}^{({\rm{Vm}}-{{\rm{V}}}_{1/2})/{\rm{k}}}}$$

where *I* is the peak current, *I*_max_ is the maximal peak current from a holding potential of −90 mV, V_1/2_ is the potential at which half current was inactivated, Vm is the potential of the conditioning pulse and *k* is the slope factor.

### Statistics and reproducibility

Data analysis and fitting procedures were performed with Prism v8 (GraphPad Software, Inc, San Diego, CA). Data are presented as the mean ± S.E.M. When data followed a normal distribution, significant differences were assessed with Student *t*-tests for multiple comparisons. In other cases, significant differences were assessed with Mann-Whitney tests. For biochemical assay of acetylcholinesterase activity, the statistical analysis was made by using Analysis of Variance (one-way ANOVA). Statistical analysis was expressed as non-significant for *p* > 0.05 and significant for *p < 0.05, **p < 0.01 and ***p < 0.001.

### Calcium imaging

Neurons were isolated, as already mentioned above. The cells were washed two times in saline and incubated in the dark with 2 µM Fura-2 pentakis (acetoxy-methyl) ester (Fura-2 AM) for 40 min at 29 °C. After loading, cells were washed two times in saline. The glass coverslips were then mounted in a recording chamber (Warner Instruments, Hamden, CT, USA) connected to a gravity perfusion system allowing drug application. Imaging experiments were performed with an inverted Nikon Eclipse Ti microscope (Nikon, Tokyo, Japan) equipped with epifluorescence. Excitation light was provided by a 75-W integral xenon lamp. Excitation wavelengths (340 nm and 380 nm) were applied using a Lambda DG4 wavelength switcher (Sutter instrument, Novato, CA, USA). Images were collected with an Orca-R2 CCD camera (Hamamatsu photonics, Shizuoka, Japan) and recorded on the computer with Imaging Workbench software (version 6, Indec BioSystems Santa Clara, CA, USA). Experiments were carried out at room temperature. Intracellular calcium level was expressed as the ratio of emitted fluorescence (340/380 nm).

### Biochemical assay of acetylcholinesterase activity

Neuronal Acetylcholinesterase (AChE) activity was determined according to the spectrophotometric method previously described^75^. The AChE source was the isolated neuronal cells (10 heads from Kisumu or AcerKis *An. gambiae* dissociated in 1 mL of the mosquito saline buffer containing: 130 mM NaCl, 2.5 mM KCl, 5 mM CaCl2, 3 mM MgCl_2_, 5 mM HEPES, 50 mM sucrose, 5% fetal bovine serum, 50 μg/mL streptomycin et 50UI/mL penicillin, pH was adjusted to 7.2 with NaOH. Biochemical assays were carried out using 90 µL of AChE source, 100 µL of 1 mM acetylthiocholine iodide (ATC) (Sigma-Aldrich, St Quentin-Fallavier, France) and 100 µL of 1 mM dithio-dinitrobenzoate (DTNB) (Sigma-Aldrich, St Quentin-Fallavier, France) in 0.1 M sodium phosphate buffer (pH 7.4). After 2 h of color development, the reaction was monitored at 405 nm, with a Bio-Tek EL808 Ultra Microplate reader (Biotek Instruments). The AChE activity from AcerKis neuronal cells was expressed as relative activity compared to Kis one. Data were analyzed with GraphPad Prism v8 (GraphPad software, Inc) and displayed as mean ± S.E.M.

### Transcriptional analysis by RT-qPCR

Neuronal cells were dissociated from 10 emerging females mosquito heads. Neuronal suspension was aspirated to remove cell debris and centrifuged at 13,000 *g* for 10 min. Pellets were stored at −80 °C until use. Total RNA were extracted from frozen pellets with Nucleospin^®^ RNA Plus kit (Macherey Nagel) and eluted in 60 µL of RNase- and DNase-free water. The total mRNA was precipitated with glycogen, sodium acetate and ethanol at −80 °C overnight. The pellet resulting from centrifugation (12,000 *g* for 15 min at 4 °C) was washed twice with 75% ethanol, air dried, resuspended in 20 μL water. The first-strand cDNA was synthesized in 20 µL reaction volumes using 1.5 µg of total RNA, 500 ng of oligo(dT) primers and the RevertAid H Minus First Strand cDNA Synthesis Kit (Thermo Scientific). Quantitative real-time PCR was performed in 20 µL reaction volumes with MESA GREEN qPCR MasterMix Plus for SYBR^®^ Assay I Low ROX (Eurogentec), 1 µM of primers and 5 µL of 1:20 diluted cDNA. Reactions were run on a CFX Connect Real-Time PCR Detection System (Bio-rad) using the following program: initial step at 95 °C for 5 min followed by 40 cycles of 95 °C for 15 s, 62 °C for 30 s and 72 °C for 1 min. After the PCR reactions were complete, melt curve analyses were carried out. Four replicates were obtained for each strain. The expression level of Ca_v_1, the α1 subunit of the voltage-gated Ca^2+^ channel, gene was normalized to the expression level of *rps7* or *actin* reference genes and relative expression levels were calculated according to the 2^−ΔCt^ method^76^. Primers are indicated in Supplementary Table 2. The PCR efficiencies were over 94%, which is acceptable for reliable real-time PCR quantification. For statistical analysis, one-way ANOVA was performed with 95% confidence intervals using GraphPad Prism v8.

### Larval bioassays

The bioassays were carried out using clothianidin prepared in absolute ethanol and stored at 4 °C throughout the experimentation. Insecticide was serially diluted from a 1000 mg/L stock solution in absolute ethanol in series by transferring 2 mL insecticide solution in 18 mL absolute ethanol. The larval bioassays were performed using a standard protocol described by the World Health Organization^77^. Each bioassay was repeated three to four times using late third- and early fourth-instar larvae of Kis, AcerKis and KdrKis. For each bioassay, 25 larvae of each strain were transferred to cups containing 99 mL of distilled water. For each bioassay, we used four cups per concentration (100 larvae) and seven concentrations of each insecticide in a range between 0 and 100% mortality. One milliliter of clothianidin, at the desired concentration, was added to the cups. Negative controls with 1 mL of absolute ethanol were performed for each test. Each bioassay was maintained at 28 °C. Larval mortality was recorded after 24 h and 48 h exposure. Tetramin® fish food powder was added in the cups after 24 h mortality recording. Larval mortality was corrected by the Abbott formula to take into account control mortality (<10%), and data were analyzed by the log-probit method of Finney with the R software using the R script BioRssay^77–80^. The programmed script uses the iterative method of maximum likelihood to fit a linear regression between the logarithm of concentration and the probit of mortality. The goodness-of-fit was estimated by a weighted chi-squared test. It also estimates the lethal concentrations and the slope of the regression lines with their confidence intervals.

### Reporting summary

Further information on research design is available in the Nature Research Reporting Summary linked to this article.

## Supplementary information

Supplementary Information

Supplementary Data 1

Reporting Summary

Description of Additional Supplementary Files

## Data Availability

The accession number used for the RT-qPCR analysis were obtained from GenBank (*rps7* gene, GenBank accession number XM_314557.3; *actin* gene, GenBank accession number XM_315271.4; *Cav1* gene, GenBank accession number EF595743.1). All other data needed to evaluate the conclusions in the paper are present in the paper or the Supplementary Information. All source data generated and analyzed during the current study are available in Supplementary Data 1. Data can also be obtained from the corresponding author upon reasonable request.
