# Supplementary material for: Compensatory mechanisms in resistant Anopheles gambiae AcerKis and KdrKis neurons modulate insecticide-based mosquito control
Source: Commun Biol. 2021 Jun 2;4:665. doi: 10.1038/s42003-021-02192-0 (PMC8172894; doi:10.1038/s42003-021-02192-0)
Supplement: Supplementary file 1 — Supplementary Information [file 42003_2021_2192_MOESM1_ESM.pdf]

SUPPLEMENTARY INFORMATIONS :

Compensatory mechanisms in resistant *Anopheles gambiae* AcerKis and KdrKis neurons modulate insecticide-based mosquito control

Stéphane Perrier[1], Eléonore Moreau[1], Caroline Deshayes[1], Marine El-Adouzi[1], Delphine Goven[1], Fabrice Chandre[2] & Bruno Lapied[1*]

[1]Univ Angers, INRAE, SIFCIR, *SFR* QUASAV, F-49000 Angers, France

[2]MIVEGEC, UMR IRD 224-CNRS 5290-Université de Montpellier, 911 avenue Agropolis, 34394 Montpellier Cedex 05, France.

*Corresponding author: Prof. B. Lapied, laboratoire SiFCIR, UFR Sciences, 2 boulevard Lavoisier, 49045 Angers, cedex, France; E-mail adress : bruno.lapied@univ-angers.fr

Supplementary Table 1 Voltage dependence of activation and inactivation of inward sodium and calcium currents in Kis and KdrKis neurons.

Sodium currents		Kis neurons	KdrKis neurons
Activation	$V_{0.5}$1 (mV)	-55,49 ± 1,99 (n=8-10)	-55,73 ± 1,78 (n=5-7)
	k1 (mV)	6,71 ± 0,84 (n=8-10)	5,73 ± 1,64 (n=5-7)
	$V_{0.5}$ 2 (mV)	-16,68 ± 1,86 (n=8-10)	-21,31 ± 1,62 (n=5-7)
	k2 (mV)	5,78 ± 0,56 (n=8-10)	4,11 ± 0,64 (n=5-7)
Inactivation	$V_{0.5}$ (mV)	-48,50 ± 3,55 (n=5-8)	-46,99 ± 4,40 (n=2-4)
	k (mV)	10,56 ± 1,07 (n=5-8)	14,87 ± 1,88 (n=2-4)
Calcium currents		Kis neurons	KdrKis neurons
Inactivation	$V_{0.5}$ (mV)	-24.18 ± 2.17 (n=7)	-23.03 ± 0.70 (n=4)
	k (mV	8.34 ± 2.20 (n=7)	6.35 ± 2.14 (n=4)

The number of experiments (n) are biologically independent samples.

Supplementary Table 2 Primers used for RT-qPCR analysis of voltage-gated calcium channels expression level in Kis and AcerKis neurons.

Gene	GenBank Accession number	Forward primer (5’ to 3’)	Reverse primer (3’ to 5’)
rps7	XM_314557.3	GCCATCCTGGAGCTGGAGATGAA	GACGGGTCTGTACCTTCTGGA
actin	XM_315271.4	CAGTCCAAGCGTGGTATCCTCA	CTCAGTCAGCAGGACTGGGTGCT
Ca_v1	EF595743.1	TTCGATGAAGTTTTACCGCCAAC	TGCTAGAACCACCCTTCATTCC

Supplementary Figure 1

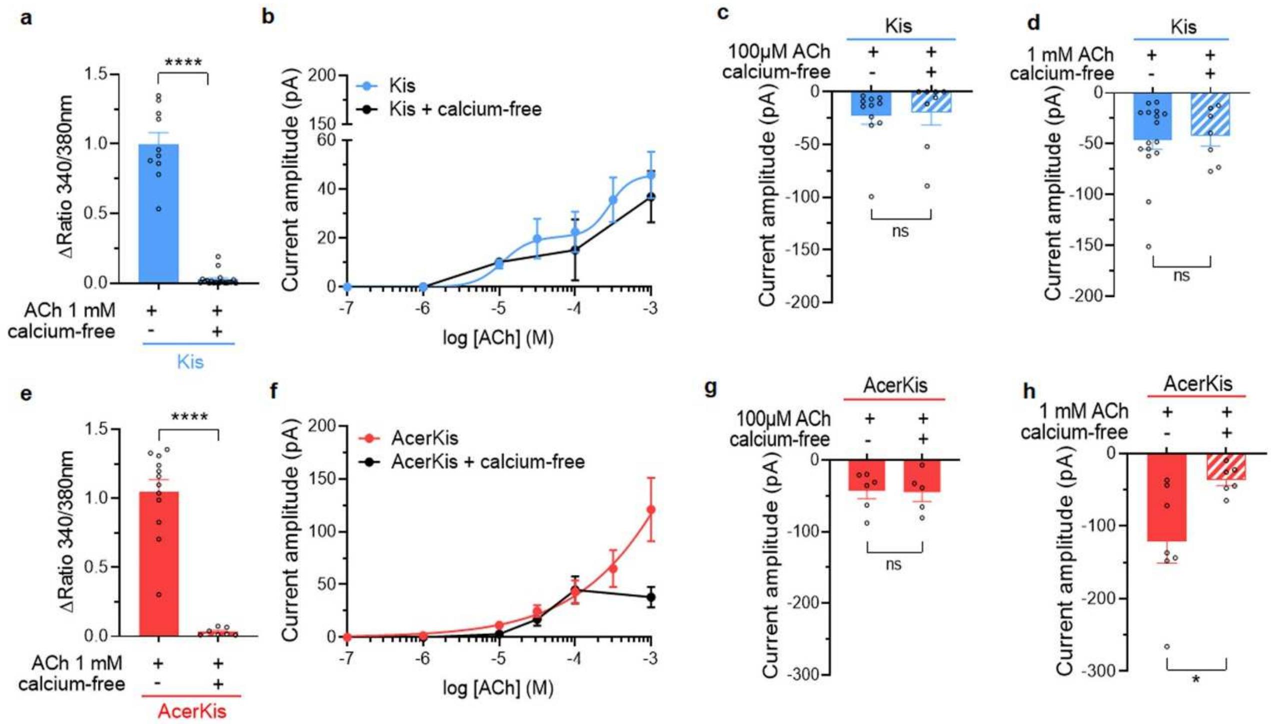

Supplementary Figure 1 Calcium free solution has different effects on the ACh-induced inward current recorded in neurons isolated from Kis and AcerKis strains. a, e Comparative histogram illustrating the effects of calcium free solution on the ACh-induced $[Ca^{2+}]_i$ rise (expressed as ratio 340/380 nm) measured in Fura-2-loaded Kis (**a**) and AcerKis (**e**) neuron cell bodies. In both cases, calcium free solution strongly decreases the intracellular calcium elevation evoked by ACh in Kis (**a**, control, n=10; in the presence of calcium free soltution, n=20) and AcerKis neurons (**c**, control, n=12; in the presence of calcium free solution, n=6). Bars represent mean ± S.E.M.. The statistical test used was Student unpaired t-test, ****, p<0.0001. **b, f** Superposed semi-logarithmic dose-response curves for the ACh-induced currents recorded in control and in the presence of calcium-free solution at a holding potential of -50mV in isolated neurons from mosquito strains Kis (**b**, control, n=5-16 ; in the presence of calcium-free solution, n=5-6) and AcerKis (**f**, control, n=3-14 ; in the presence of calcium-free solution, n=5-9), as indicated in the graph. Data are mean ± S.E.M.. Note that calcium -free solution only reduced the ACh-induced current amplitude for concentrations higher that 100µM

in AcerKis neurons. **c, d** Comparative histogram illustrating the lack of effect of calcium-free solution on the ACh-induced current amplitudes recorded at two different concentrations in Kis neurons (**c**, 100µM ACh, control n=11 ; in the presence of calcium-free solution, n=9 and **d**, 1mM ACh, control n=16 ; in the presence of calcium-free solution n=7). Bars represent mean ± S.E.M. The statistical test used was Student unpaired t-test, ns, non-significant. **g, h** Comparative histogram illustrating the effect of calcium-free solution on the ACh-induced current amplitudes recorded at two different concentrations in AcerKis neurons (**g**, 100µM ACh, control, n=6 ; in the presence of calcium free solution, n=5 and **h**, 1mM ACh, control n=7 ; in the presence of calcium free solution, n=6). Bars represent mean ± S.E.M. The statistical test used was Student unpaired t-test, ns, non-significant, *, $p<0.05$. In all cases, the number of experiments (n) are biologically independent samples.
