# Supplementary material for: Compensatory mechanisms in resistant Anopheles gambiae AcerKis and KdrKis neurons modulate insecticide-based mosquito control
Source: Commun Biol. 2021 Jun 2;4:665. doi: 10.1038/s42003-021-02192-0 (PMC8172894; doi:10.1038/s42003-021-02192-0)
Supplement: Supplementary file 3 — Reporting Summary [file 42003_2021_2192_MOESM3_ESM.pdf]

nature research

Corresponding author(s): Prof B. Lapied

Last updated by author(s): Mar 23, 2021

Reporting Summary

Nature Research wishes to improve the reproducibility of the work that we publish. This form provides structure for consistency and transparency in reporting. For further information on Nature Research policies, see our Editorial Policies and the Editorial Policy Checklist.

Statistics

For all statistical analyses, confirm that the following items are present in the figure legend, table legend, main text, or Methods section.

n/a	Confirmed	
☐	☒	The exact sample size (*n*) for each experimental group/condition, given as a discrete number and unit of measurement
☐	☒	A statement on whether measurements were taken from distinct samples or whether the same sample was measured repeatedly
☐	☒	The statistical test(s) used AND whether they are one- or two-sided<br>*Only common tests should be described solely by name; describe more complex techniques in the Methods section.*
☐	☒	A description of all covariates tested
☒	☐	A description of any assumptions or corrections, such as tests of normality and adjustment for multiple comparisons
☐	☒	A full description of the statistical parameters including central tendency (e.g. means) or other basic estimates (e.g. regression coefficient) AND variation (e.g. standard deviation) or associated estimates of uncertainty (e.g. confidence intervals)
☐	☒	For null hypothesis testing, the test statistic (e.g. *F*, *t*, *r*) with confidence intervals, effect sizes, degrees of freedom and *P* value noted<br>*Give P values as exact values whenever suitable.*
☒	☐	For Bayesian analysis, information on the choice of priors and Markov chain Monte Carlo settings
☒	☐	For hierarchical and complex designs, identification of the appropriate level for tests and full reporting of outcomes
☒	☐	Estimates of effect sizes (e.g. Cohen's *d*, Pearson's *r*), indicating how they were calculated

Our web collection on statistics for biologists contains articles on many of the points above.

Software and code

Policy information about availability of computer code

Data collection	Ionic currents were displayed on a computer with software control pClamp (version 10.0; Molecular Devices) connected to a digitizer (DIGIDATA 1322; Molecular Devices). For calcium imaging, images were collected with an Orca-R2 CCD camera (Hamamatsu photonics, Shizuoka, Japan) and recorded on the computer with Imaging Workbench software (version 6, Indec BioSystems Santa Clara, CA, USA).
Data analysis	Data analysis and fitting procedures were performed with Prism v8 (GraphPad Software, Inc, San Diego, CA). In vivo data were analyzed by the log-probit method of Finney with the R software using the R script BioRssay.<br>Reference: Milesi P, Pocquet N, Labbé P: BioRssay: a R script for bioassay analyses. https://drive.google.com/file/d/1qMNC2EQlxBnOunuaauta1BCQcLesnrFX/view

For manuscripts utilizing custom algorithms or software that are central to the research but not yet described in published literature, software must be made available to editors and reviewers. We strongly encourage code deposition in a community repository (e.g. GitHub). See the Nature Research guidelines for submitting code & software for further information.

Data

Policy information about availability of data

All manuscripts must include a data availability statement. This statement should provide the following information, where applicable:

- Accession codes, unique identifiers, or web links for publicly available datasets
- A list of figures that have associated raw data
- A description of any restrictions on data availability

All data suporting the findings of this study are available from the corresponding author upon reasonable request. All accession codes and web links are provided in the manuscript.

Field-specific reporting

Please select the one below that is the best fit for your research. If you are not sure, read the appropriate sections before making your selection.

☒ Life sciences ☐ Behavioural & social sciences ☐ Ecological, evolutionary & environmental sciences

For a reference copy of the document with all sections, see nature.com/documents/nr-reporting-summary-flat.pdf

Life sciences study design

All studies must disclose on these points even when the disclosure is negative.

Sample size	All information regarding the item Sample size are described in details in the method section of the submitted manuscript.
Data exclusions	All information regarding the item data exclusions are described in details in the method section of the submitted manuscript.
Replication	No attempt of replication of the reported study as a whole has been made, yet the study itself includes replicates for each experiments according to different experimental conditions (see methods).
Randomization	Randomization is not relevant in our study as all work was direct experimental design and all output used in each set of experiments.
Blinding	Blinding was not relevant in our study

Reporting for specific materials, systems and methods

We require information from authors about some types of materials, experimental systems and methods used in many studies. Here, indicate whether each material, system or method listed is relevant to your study. If you are not sure if a list item applies to your research, read the appropriate section before selecting a response.

Materials & experimental systems

n/a	Involved in the study	
☒	☐	Antibodies
☒	☐	Eukaryotic cell lines
☒	☐	Palaeontology and archaeology
☐	☒	Animals and other organisms
☒	☐	Human research participants
☒	☐	Clinical data
☒	☐	Dual use research of concern

Methods

n/a	Involved in the study	
☒	☐	ChIP-seq
☒	☐	Flow cytometry
☒	☐	MRI-based neuroimaging

Animals and other organisms

Policy information about studies involving animals; ARRIVE guidelines recommended for reporting animal research

Laboratory animals	Three Anopheles gambiae female mosquito strains (Kis, AcerKis and KdrKis) were used in this study. Eggs were provided by MIVEGEC laboratory (UMR IRD-CNRS-Montpellier University) from Montpellier, France. Mosquitoes were reared in an 80% humidity and 28°C temperature environment with a 12h light/dark photo-cycle.
Wild animals	not relevant
Field-collected samples	not relevant
Ethics oversight	not relevant

Note that full information on the approval of the study protocol must also be provided in the manuscript.
