# Supplementary material for: Compensatory mechanisms in resistant Anopheles gambiae AcerKis and KdrKis neurons modulate insecticide-based mosquito control
Source: Commun Biol. 2021 Jun 2;4:665. doi: 10.1038/s42003-021-02192-0 (PMC8172894; doi:10.1038/s42003-021-02192-0)
Supplement: Supplementary file 4 — Description of Additional Supplementary Files [file 42003_2021_2192_MOESM4_ESM.pdf]

Description of Additional Supplementary Files

File name: Supplementary Movie 1

Description: All source data generated and analysed during the current study.
